# Genetic program activity delineates risk, relapse, and therapy responsiveness in multiple myeloma

**DOI:** 10.1038/s41698-021-00185-0

**Published:** 2021-06-28

**Authors:** Matthew A. Wall, Serdar Turkarslan, Wei-Ju Wu, Samuel A. Danziger, David J. Reiss, Mike J. Mason, Andrew P. Dervan, Matthew W. B. Trotter, Douglas Bassett, Robert M. Hershberg, Adrián López García de Lomana, Alexander V. Ratushny, Nitin S. Baliga

**Affiliations:** 1 Institute for Systems Biology, Seattle, WA USA; 2 Bristol-Myers Squibb, Summit, NJ USA; 3 Sage Bionetworks, Seattle, WA USA; 4 Celgene Institute for Translational Research Europe (CITRE), a Bristol-Myers Squibb Company, Summit, NJ USA; 5 Celgene Corporation, Seattle, WA USA; 6 Departments of Biology, Microbiology, and Molecular Engineering Sciences, University of Washington, Seattle, WA USA; 7 Lawrence Berkeley National Labs, Berkeley, CA USA

**Keywords:** Myeloma, Systems biology, Cancer genomics, Target identification, Risk factors

## Abstract

Despite recent advancements in the treatment of multiple myeloma (MM), nearly all patients ultimately relapse and many become refractory to multiple lines of therapies. Therefore, we not only need the ability to predict which patients are at high risk for disease progression but also a means to understand the mechanisms underlying their risk. Here, we report a transcriptional regulatory network (TRN) for MM inferred from cross-sectional multi-omics data from 881 patients that predicts how 124 chromosomal abnormalities and somatic mutations causally perturb 392 transcription regulators of 8549 genes to manifest in distinct clinical phenotypes and outcomes. We identified 141 genetic programs whose activity profiles stratify patients into 25 distinct transcriptional states and proved to be more predictive of outcomes than did mutations. The coherence of these programs and accuracy of our network-based risk prediction was validated in two independent datasets. We observed subtype-specific vulnerabilities to interventions with existing drugs and revealed plausible mechanisms for relapse, including the establishment of an immunosuppressive microenvironment. Investigation of the t(4;14) clinical subtype using the TRN revealed that 16% of these patients exhibit an extreme-risk combination of genetic programs (median progression-free survival of 5 months) that create a distinct phenotype with targetable genes and pathways.

## Introduction

Multiple myeloma (MM) is a cancer of malignant plasma cells in the bone marrow (BM) that has a prevalence of ~86,000 new cases per year^1^. Several clinical subtypes of MM have been established on the basis of characteristic cytogenetic features, including various translocations, gain or loss of chromosomal arms, deletion of specific chromosomes, and hyperdiploidy^2–4^. Accordingly, MM is a complex disease of great heterogeneity that exhibits subtype-specific drivers of progression^5,6^. Efforts to better characterize the biology and therapeutic vulnerabilities of MM have increased exponentially in recent years, as can be seen from the number of research articles, clinical trials, and public availability of matched genomic, transcriptomic, and patient data. However, the myriad combinations of chromosomal aberrations and somatic mutations, coupled with the complex dependence of MM progression on the BM microenvironment, have precluded a mechanistic understanding of the disease on a patient-specific level.

If the disease biology of an individual patient can be sufficiently well characterized from experimental assays, it is conceivable that they can be assigned the best available therapies and manage their cancer like a chronic illness with much-improved outcomes. However, a detailed map of the underlying biology of MM is necessary to translate the data collected from a patient into personalized recommendations for therapy. The development of such a map is complicated by the great degree of heterogeneity MM exhibits, including subtypes at the levels of gene expression, gene mutations, chromosomal abnormalities, and clinical outcomes. Before we can establish an era of personalized medicine for all MM patients, we must understand how the subtypes at these different levels relate to one another mechanistically, and which of these features are most important for determining the risk of disease progression. Moreover, we must characterize the biological changes that drive escape from therapy and the onset of relapse-refractory disease. Once we understand the subtype-specific drivers of disease progression and biology of relapse, we can rationalize and test which therapies are most appropriate for which patient subtypes.

We hypothesized that a causal-mechanistic (CM) transcriptional regulatory network (TRN) would provide a robust framework to establish the desired map of underlying biology that relates mutations, gene expression, and clinical outcomes in a comprehensible and actionable way. A CM TRN is a network inferred from multi-omics data that identifies mechanisms by which some genes (e.g., transcription factors (TFs)) regulate the expression of other genes and reveals how different mutations and chromosomal aberrations dysregulate these processes, thereby leading to hallmarks of cancer^7^. Patterns in the CM TRN can be related to clinical outcomes in order to elucidate the biological context of a heterogeneous disease. Thus, the different levels of MM heterogeneity can all be linked to one another in a CM TRN. However, there are several challenges to the development and application of CM TRNs, including spurious correlations that arise from the high dimensionality of gene expression data, difficulty of detecting rare features such as condition-specific regulatory mechanisms, complexity of inferring causal events, and the requirement of efficient computational algorithms^8–11^. Moreover, there is no generally accepted protocol to infer which features of the network are activated or deactivated in an individual patient. This is of critical importance because the CM TRN represents possible biological mechanisms across all subtypes of a disease, such that each individual will only exhibit activity in a subset of the network features.

Several methods have been developed for TRN inference, including GENIE3^12^ (and the closely related GRNboost^13^), ARACNe^11^, CLR^14^, and cMonkey^8^. Some network inference methods (i.e., mechanistic inference methods) incorporate orthogonal biological evidence, such as the presence of TF binding sites in the promoter regions of target genes, to increase the confidence of inferred regulatory relationships. To complete the construction of a CM TRN, a causal inference method can be applied to a TRN and matched mutation data. Causal inference can be performed either by comparing the likelihood of different structural equation models (e.g., NEO^15^, FINDr^16^), identifying predictive features in a machine-learning model fit to gene expression, or clustering mutations according to common occurrence in a predefined functional network (NBS^17^). The merits and weaknesses of these approaches and a description of the specific causal inference algorithm used in this work are detailed in the Supplementary information.

Although gene expression networks have previously been derived to study MM^18–20^, a CM TRN that elucidates causal flows from mutations to regulators to co-regulated genes across MM subtypes has not yet been established. In this work, we present a new method called mechanistic inference of node-edge relationships (MINER) to construct a CM TRN from multi-omics and clinical outcomes data, infer patient-specific network activity, and identify subtype-specific mechanisms that are likely to predispose resistance or susceptibility to a given therapy. We apply this method to better characterize MM, with the specific goal of elucidating the underlying biology of high-risk clinical subtypes and the changes that occur at relapse.

## Results

### MINER pipeline infers CM TRN of MM

**Fig. 1:**
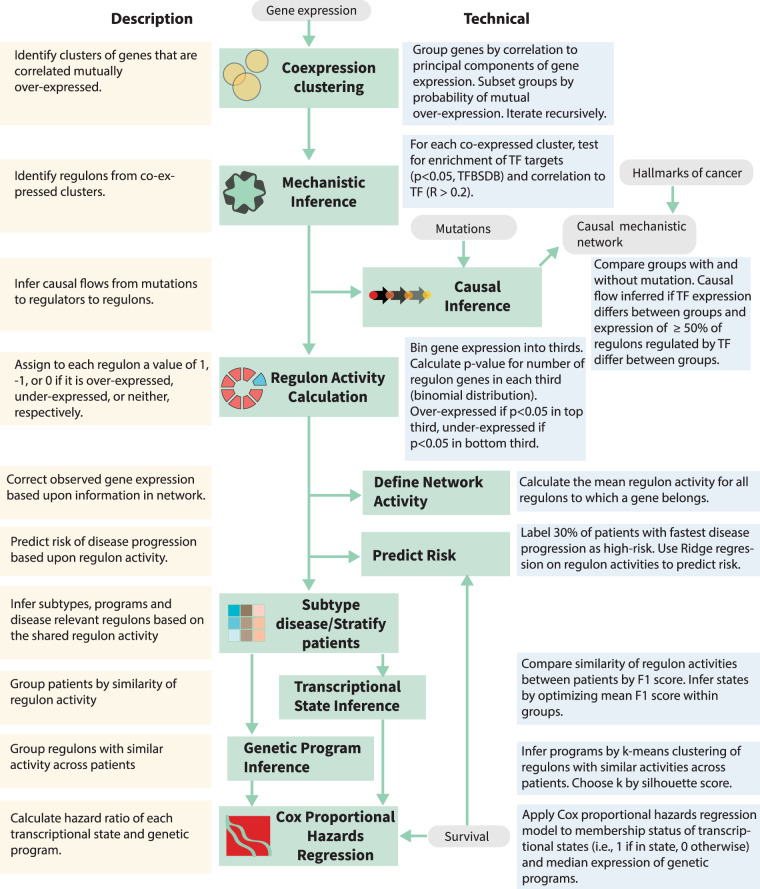
Mechanistic inference of node-edge relationships (MINER). MINER applies a gene expression clustering algorithm and gold-standard gene interaction databases to infer sets of co-regulated genes called regulons. Each regulon has an associated regulator and edge direction (i.e., activation or repression), as well as an activity in each sample (e.g., overexpressed, underexpressed, etc.). When the coordinated activity of regulons changes in the context of a mutation, a causal relationship is inferred through the associated regulator. A causal and mechanistic transcriptional regulatory network is generated by evaluating the influence of all potential causes (e.g., mutations, translocations, copy-number abnormalities, etc.) on all regulons. Prediction of phenotypes such as responsiveness to therapy or risk of disease progression is achieved by training machine-learning algorithms on regulon activities. The predictive signatures are then placed into a meaningful biological context by identifying their associated mechanisms and putative causes within the network.

We developed the MINER pipeline to infer TRNs from gene expression data and apply them to the characterization and prediction of phenotypes. MINER builds upon our previous work with the SYstems Genetics Network AnaLysis (SYGNAL) pipeline insofar as it enables the same core functionalities of mechanistic and causal inference, but does so with a new suite of algorithms that enable new applications in the network-based prediction of clinical outcomes (Fig. 1)^7^. Inference of the TRN begins by clustering gene expression data into coherent sets of genes that share a binding site for a TF or microRNA (miRNA) according to gold-standard binding-site database information (e.g., transcription factor binding-site database—TFBSDB). By default, the cluster of genes and the corresponding regulator must also be correlated (or anticorrelated) to one another. Many TFs are regulated at the post-transcriptional level, however, so we only enforce a mild correlation (*R* > 0.2), and this restriction can be lifted entirely if it proves too stringent, e.g., in single-cell analysis. The combination of a coherently expressed set of genes and the associated regulator whose binding site they share represent discrete units, called *regulons*, from which the TRN is assembled. Once the regulons have been discovered, a new causal inference algorithm (see “Methods” and Supplementary information) measures the impact of a mutation on a regulator by comparing the changes to downstream regulon activities with what could be expected by random chance (Supplementary Fig. 1). Once a MINER TRN has been inferred from the data of a patient cohort, new samples can be analyzed to uncover the disease-relevant modules that are over- or under-active in an individual patient.

Application of MINER to the Multiple Myeloma Research Foundation (MMRF) Interim Analysis 12 (IA12) dataset successfully generated a CM TRN of MM. The network features 15,192 genes partitioned into 1233 coexpression clusters (i.e., without inferred co-regulation), 8549 genes partitioned into 3203 co-regulated modules (called *regulons* herein) that are regulated by 392 unique TFs, and 124 causal drivers, including somatic mutations, translocations, and cytogenetic abnormalities. We note that miRNA-sequencing (miRNA-seq) was not performed in the MMRF CoMMpass study, so the inference of miRNA regulation was limited to the analysis of target gene expression (see “Methods”) and was, therefore, less robust than TF analysis. The results of our miRNA inference are included in the online portal, but the remainder of this manuscript will focus on regulation mediated by TFs.

In total, the MINER network comprises 13,587 unique CM flows—links from mutations to regulators to co-regulated genes. Every mutation or chromosomal aberration that occurred in at least 2% of the patient population is represented within the network, such that its inferred causal effect on the regulation of gene expression is described. This includes a recapitulation of the known transcriptional effects, such as the upregulation of *NSD2* by t(4;14), *MAF* by t(14;16), and *CCND1* by t(11;14). Moreover, the regulons that associate with the risk of disease progression in the CM TRN are enriched with upstream mutations that have previously been implicated in MM. We tested this by filtering the network to only include regulons whose activity was greater than a minimum Cox hazard ratio (HR) threshold (see online portal for detailed analysis). As we increased the threshold from 2 to 6, the percent of upstream causal mutations that were validated as associated to MM in the literature monotonically increased (|HR| > 2: 31% validated; |HR| > 3: 33% validated; |HR| > 4: 36% validated; |HR| > 5: 41% validated; |HR| > 6: 46% validated). The complete MINER network is provided as an interactive web portal and available for download at https://myeloma.systemsbiology.net/.

We benchmarked the performance of MINER against SCENIC^13^, which is the closest alternative mechanistic inference method to our knowledge. Although SCENIC was originally developed for single-cell analysis, its algorithms also work for bulk RNA-seq data (e.g., its network inference algorithm was originally developed for bulk RNA-seq), and the mechanistic inference pipeline design is an even closer match to MINER than is SYGNAL. In particular, SCENIC identifies regulons from gene expression data and TF binding-site information and provides a measure of each regulon’s activity in each sample. We prioritized benchmarking metrics that address evidence for co-regulation and quantify the amount of information lost when moving from gene expression to regulon activity. We evaluated the evidence for co-regulation by measuring the regulon coherence (i.e., variance of gene expression) and the percent of genes sharing a regulator binding site in the promoter region according to the TFBSDB^7^. We chose the TFBSDB because it includes evidence for chromatin accessibility in addition to the presence of TF binding site motifs. The preservation of information upon reducing gene expression to regulon activity was quantified by Spearman’s rank correlation coefficient of pairwise Euclidean distances between samples in the gene expression space versus regulon activity space^21^.

Supplementary Fig. 2 shows that MINER outperforms SCENIC in all three benchmark measures of mechanistic inference. Because MINER uses TFBSDB as a reference database, it is not surprising that 100% of MINER regulons are enriched (i.e., hypergeometric *p* value < 0.05) with TFBSDB targets. However, <40% of SCENIC regulons are significantly enriched with TFBSDB targets, which may result from the inclusion of genes that have a TF binding sequence but no chromatin accessibility. Permutation analysis showed that only 47% of SCENIC regulons had gene expression variance significantly lower than expected by random chance, compared to 78% for MINER. Finally, the preservation of gene expression topology measured by Spearman’s rank correlation coefficient was significantly greater (*p* = 3.5 × 10^−289^, Wilcoxon’s rank-sum test) in regulons discovered by MINER (*R* = 0.84 ± 0.06) versus SCENIC (*R* = 0.36 ± 0.09).

**Fig. 2:**
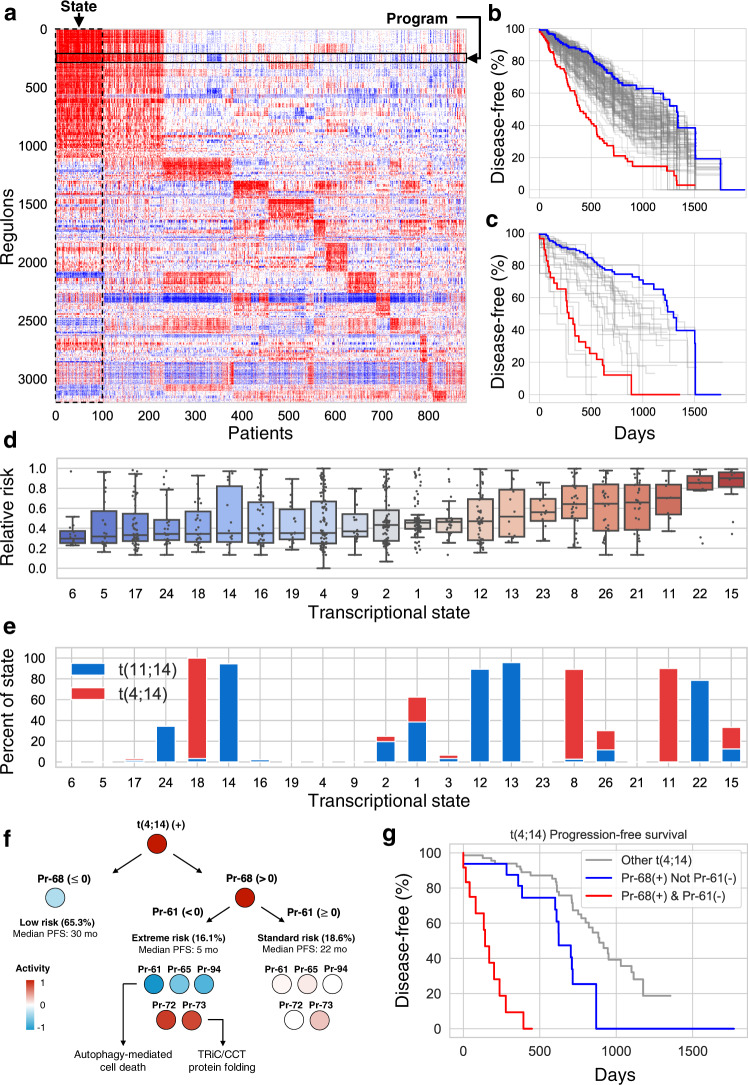
Programs and states. **a** Heatmap of regulon activity across patients reveals distinct transcriptional states wherein patients have very similar regulon activity across the entire network. **b**, **c** Kaplan–Meier survival curves relating overexpression of each program (**b**) and state (**c**) to the observed progression-free survival in the CoMMpass study. The highest risk (red) and lowest risk (blue) programs are highlighted in (**b**). The four lowest risk (blue) and three highest risk (red) states are pooled to generate the highlighted curves in (**c**). **d** The risk distribution of each state according to the GuanRank of the survival data. States with fewer than five patients represented in the survival data were omitted. Center line, median; box limits, upper and lower quartiles; whiskers, 1.5× interquartile range. **e** Patients with t(4;14) or t(11;14) fall into several distinct transcriptional states with varying risk of disease progression. **f** Program activity sub-stratifies t(4;14) into standard and extreme-risk groups. **g** Kaplan–Meier plot of substratified t(4;14) groups.

The improved topology preservation by MINER versus SCENIC can be further appreciated when viewing the regulon activity heatmaps. For each regulon, MINER classifies its status as overexpressed, underexpressed, or neither using a *p* value cutoff of 0.05 for each patient sample as described in the “Methods” section. A heatmap of the regulon activities across all patient samples reveals distinct patterns of co-regulated gene expression (Fig. 2a). The presence of activated and repressed regulons and the clear existence of patient subtypes (i.e., transcriptional states) can be seen in the MINER regulons, but not in those discovered by SCENIC (Supplementary Fig. 3). Inspection of a representative TF, *MAF*, and its regulons identified by SCENIC versus MINER sheds light on the regulon-level differences in activity and topology preservation (Supplementary Fig. 4). SCENIC identifies a single regulon with all inferred target genes of *MAF* that are activated (i.e., no repressed target genes identified). MINER, on the other hand, identifies several distinct *MAF* regulons, some of which are activated by *MAF* and others that are repressed. The different regulons are distinguished by distinct activity patterns across samples (i.e., biclustering).

### MM gene expression exhibits a hierarchy of genetic programs and transcriptional states

Clusters of regulons, called *genetic programs* (or simply *programs*) herein, were observed to have similar activity across patient samples (Fig. 2a). In total, we discovered 141 programs, with an average of 90 unique genes and 21 unique TFs (i.e., 21 distinct regulons) per program. We evaluated the coherence of these genetic programs in two independent test datasets of MM (GSE24080^22^ and GSE19784^23^) by comparing the variance of the genes in each program against that of random selections of the same number of genes (500 permutations). 94.3% of the programs were coherent (variance < random, *p* < 0.05) in GSE24080, and 92.2% were coherent in GSE19784, despite the fact that the training data (MMRF IA12) was collected via RNA-seq and the validation was microarray-based. The high degree of program coherence in multiple test datasets validates the generality of genetic programs discovered by our approach.

Regulon activity was also observed to cluster patients into subtypes of similar overall gene expression, which we call *transcriptional states* (Fig. 2a). Using our default clustering algorithm (see “Methods” section), we discovered 25 distinct transcriptional states that accounted for 95% of the total patient population. The remaining 5% of patients did not match any of the states sufficiently well. We tested alternative clustering algorithms and found similar results (Supplementary Fig. 5). The presence of individual mutations or chromosomal abnormalities was insufficient to predict a patient’s transcriptional state. The only mutations that occurred in at least 80% of the patients in a transcriptional state were t(4;14) and t(11;14). However, these translocations occurred in several states, so their presence alone did not determine to which state a patient belonged. Gene set enrichment analysis of the differentially expressed genes in each transcriptional state indicates that differences in the activity levels of cell cycle progression and immune response pathways are responsible for the classification of t(4;14) and t(11;14) patients into several distinct states (Supplementary Fig. 6).

### Mutations and environmental factors drive the states via TF networks

In order to better understand the drivers of a transcriptional state, we considered the use case of transcriptional state 15 (TS-15)—the highest risk state. We determined the set of activated and repressed regulons characteristic of TS-15 by performing differential regulon expression analysis (see “Methods”). Because each regulon is matched to one regulator, this process also provides a list of TFs that regulate the characteristic regulons of TS-15. We used this list of TFs to generate a TF-TF network (Supplementary Fig. 7; see “Methods”) in order to identify putative master regulators of TS-15. By linking the TFs to upstream causal mutations in the CM TRN, we found that specific TF-TF subnetworks were activated by different mutations (Supplementary Fig. 8). For example, the most common mutations in the patients of TS-15—*NRAS* (48%), Amp 1q (47%), and *TP53* (26%)—directly activated complementary TF-TF subnetworks; the consequences of which propagated indirectly through the dense TF-TF network to generate the same global pattern of gene expression characteristic to TS-15.

In addition to mutations, we note that microenvironment features can be causes of specific gene expression profiles (e.g., by activating TF-TF networks). MINER enables gene set enrichment analysis of the differentially expressed genes in each state with any reference database as a means to identify potential nonmutation causes of gene expression patterns. The results of gene set enrichment analysis of TS-15 using the Molecular Signatures Database Hallmark pathway database as an example are included in Supplementary Fig. 6. The hallmark targets of *E2F* and *MYC* are highly enriched in TS-15, which is consistent with the presence of *E2F1* and *MYC* in the TS-15 TF-TF network as influential activators of many other TFs.

### The CM TRN enables robust risk stratification

We tested the ability of a patient’s network status (i.e., the list of which regulons are activated and deactivated) to predict the risk of disease progression using Ridge regression trained on the regulon activities of MMRF IA12. The predictive performance was evaluated in two microarray-based validation datasets of MM (GSE24080 and GSE19784) by first applying our method of calculating discrete activities for all regulons in each patient of those datasets and then applying the predictor. A very strong performance was observed in both datasets (Table 1), with validation AUC (i.e., area under the receiver operating characteristic curve) values of 0.70 in GSE24080 and 0.71 in GSE19784. This performance is on par with the best predictors available for MM^24^ and has the benefit of associating mechanisms and upstream causes to the predictive features. Thus, both the method of inferring the status of the CM TRN in individual patients and the utility of the CM TRN status as a predictor of risk for disease progression were validated in these independent datasets.

**Table 1:** Results of Ridge regression using regulon activity as features.

Dataset	*N*	Median PFS	AUC	Spearman *R*	Spearman *p*	Cox HR	Cox *p*
MMRF IA12^a^	769	631	0.86	0.59	7.2E–72	19.1	5.2E–81
GSE24080	559	1288	0.70	0.26	5.4E–10	7.4	1.1E–13
GSE19784	282	782	0.71	0.35	1.4E–9	6.3	2.7E–10

*N* number of samples, the median in days, Spearman correlation is predicted risk score versus GuanRank (i.e., observed risk rank), and Cox regression is with respect to the predicted risk score.

^a^MMRF IA12 was used for training.

Next, we performed Cox proportional hazards regression on the individual programs (Fig. 2b) and states (Fig. 2c) to quantify the extent to which these features stratify risk. Both the programs and states exhibited high- and low-risk features, with the states being particularly powerful determinants of risk (Fig. 2d). These observations stand in contrast to the results of individual mutations, which showed much less significance for stratifying risk, due in part to how infrequently most mutations appear (Supplementary Fig. 9). In general, the risk of a patient was better predicted by other patients with the same transcriptional state than by other patients with the same mutation. This is especially clear in the example of translocations t(4;14) and t(11;14), which exhibited distinct high- and low-risk transcriptional states (Fig. 2E). Moreover, network-based stratification—a method to identify subtypes of patients with functionally related mutations—did not result in groups of significantly different risk for disease progression (Supplementary Fig. 10).

### High-risk genetic program underlies proliferation and recapitulates predictive signatures

**Fig. 3:**
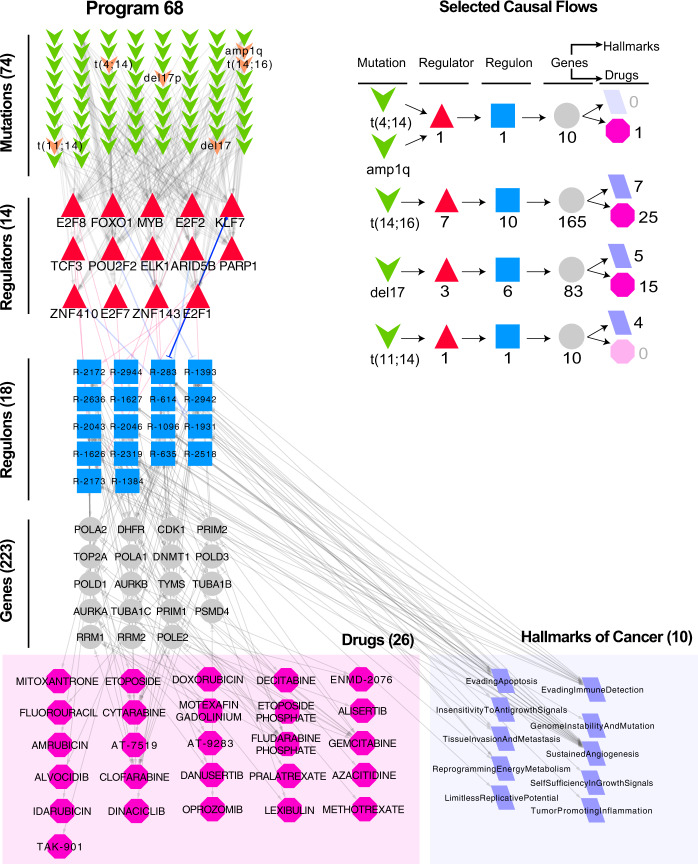
Network visualization of Pr-68. The regulons comprising program Pr-68 are represented as blue boxes with their associated regulators (red triangles) and putative causal genetic abnormalities (green chevrons). Hallmarks of cancer that are enriched in these regulons are highlighted by purple parallelograms, and drugs that target genes (gray) within these regulons are highlighted by pink octagons.

We analyzed the highest risk genetic program, Pr-68 (Cox HR = 8.8, *p* = 1.3 × 10^–18^), to better understand the mechanisms most strongly correlated to the rate of disease progression. The genes in Pr-68 were heavily enriched in DNA replication (*p* = 1.0 × 10^–25^), cell cycle (*p* = 1.0 × 10^–25^), and DNA mismatch repair (*p* = 1.0 × 10^–8^) functions, and the regulons comprising Pr-68 were enriched for ten hallmarks of cancer (Fig. 3). Thus, Pr-68 is a genetic program associated with proliferation. Interestingly, the risk stratification of Pr-68 cannot simply be explained by its cell cycle genes. In particular, the most closely matched cell cycle signatures show notably weaker risk stratification (Supplementary Fig. 11; maximum HR = 6.35, *p* = 2.18 × 10^–10^).

Several studies have identified proliferation signatures as optimal risk classifiers, but the underlying biological drivers have not been clearly established. We found that the genes of Pr-68 have an adjusted *p* value of 1 × 10^–55^ for enrichment in chromatin immunoprecipitation-seq (ChIP-seq) targets of *FOXM1*, suggesting that *FOXM1* is an important upstream regulator and potential therapeutic target. We note that *FOXM1* has been experimentally confirmed as an important target in high-risk MM^25^. *FOXM1* is most significantly activated by *E2F1* in the CM TRN (Spearman correlation: *R* = 0.77, *p* = 1.0 × 10^–172^*; E2F1 motif in FOXM1 promoter*). All high-risk subtypes, with the exception of t(4;14), were causally upstream of *FOXM1* activation via the intermediate upregulation of *E2F1* in the CM TRN.

Four published prognostic gene expression profiles of MM demonstrated significant overlap with the genes of Pr-68. We compared the gene sets of UAMS70^26^, EMC92^27^, M3CN^20^, and the Proliferation signature of Hose et al.^28^ to the Pr-68 genes and computed hypergeometric *p* values. The results, listed in Table 2, show that the overlap of all four signatures with Pr-68 was highly significant and cumulatively accounted for 108 of the 228 (47%) genes in Pr-68. Moreover, the gene *PHF19*, whose expression was identified as the best individual predictor of risk in a recent MM DREAM challenge^24^, is also a member of Pr-68. It is interesting to note that a gene module very similar to program Pr-68 can be discovered by inferring a high-confidence protein–protein interaction network via application of the STRING^29^ database to the genes that are differentially expressed with respect to risk of disease progression (Supplementary Fig. 12).

**Table 2:** Existing prognostic signatures of high-risk MM map to Pr-68.

Prognostic signature	Reference	Overlap with Pr-68	*p* value
UAMS70	Shaughnessy Jr. et al.	8/65^a^	3.0 × 10^–4^
EMC92	Kuiper et al.	17/92	2.2 × 10^–10^
Proliferation	Hose et al.	40/60	2.7 × 10^–55^
M3CN	Liu et al.	82/178	1.2 × 10^–85^
PHF19	Mason et al.	1/1	2.7 × 10^–2^

^a^Only 65/70 UAMS probes had gene names.

### Risk of disease progression is delineated by different genetic programs across myeloma subtypes

The genetic program Pr-68 has the greatest average risk across myeloma subtypes, but it is not the highest-risk program in the context of individual subtypes. The risk of each subtype is stratified by the activity of specific genetic programs, and many programs are high-risk in some subtypes but not others (Supplementary Fig. 13). However, it is interesting to note that the average expression of Pr-68 in each state is highly correlated to the average risk of the state (Supplementary Fig. 14). Nonetheless, pairwise risk analysis of different program and state activities shows that some of the highest risk combinations do not involve Pr-68 (Supplementary Fig. 15). Further relationships between regulons, programs, their inferred causal drivers, and myeloma subtypes can be explored on our online portal. Therapies associated with these risk factors are provided there and relevant literature can be directly searched from the portal.

As a case study in subtype-specific risk analysis using the CM TRN, we explored the features stratifying risk in patients bearing the t(4;14) translocation. The additional mutation which most increased risk in t(4;14) patients was *KRAS*. Patients bearing *KRAS* mutations in addition to t(4;14) were observed to be at higher risk (HR = 3.2, *p* = 1.5 × 10^–3^) than those with wild-type (WT) *KRAS*. Program expression proved to stratify risk better than any mutation in t(4;14) patients. Overexpression of programs Pr-72 (HR = 4.4, *p* = 1.2 × 10^–5^) and Pr-73 (HR = 4.6, *p* = 4.3 × 10^–6^) and underexpression of programs Pr-61 (HR = 4.5, *p* = 6.7 × 10^–6^), Pr-65 (HR = 4.3, *p* = 1.5 × 10^–5^), and Pr-94 (HR = 3.7, *p* = 2.0 × 10^–4^) were strongly associated with risk of disease progression. The t(4;14) patients stratified by these programs largely overlapped, such that individual patients tended to present many of these features simultaneously. For example, 79% of patients underexpressing Pr-61 also overexpressed Pr-72.

Interestingly, the high-risk program Pr-68 is substratified by the status of Pr-61 (Fig. 2F). Patients who underexpress Pr-61 while overexpressing Pr-68 are at extremely high risk of disease progression, with a median time to progression of ~5 months (Fig. 2G). These patients show the greatest levels of Pr-68 activation, suggesting highly proliferative disease and possible reliance on DNA-repair pathways. The remaining t(4;14) patients who overexpress Pr-68 but do not underexpress Pr-61 are at standard risk, with a median time to disease progression of ~22 months. The extreme-risk Pr-68(+)/Pr-61(−) subset exhibit significantly higher expression of the hypoxia-related genes *ENO1* and *IL-32*, and the *IL-6* signaling-related genes *IL-6R* and *IL-6ST* compared to the Pr-68(+) t(4;14) patients who did not simultaneously underexpress Pr-61. Perhaps, most importantly, this combination of program activities reflects high levels of *MYC* transcriptional activity. In particular, Pr-72 highly correlates (*R* = 0.71, *p* = 1.0 × 10^–133^) to the normalized enrichment scores of the pathway interactions database (PID) validated targets of *c-MYC* transcriptional activation (i.e., “PID MYC ACTIV PATHWAY”), and Pr-61 correlates (*R* = 0.57, *p* = 7.7 × 10^–74^) to the normalized enrichment scores of the PID validated targets of *c-MYC* transcriptional repression (i.e., “PID MYC REPRESS PATHWAY”). Moreover, Pr-72 is enriched with ChIP-seq targets of *MYC* and Pr-61 is enriched with ChIP-seq targets of *MAX* (i.e., a MYC-binding partner). Thus, *c-MYC* activation appears to be an important element of the extreme-risk t(4;14) signature.

Biological pathways were also observed to stratify risk in t(4;14) patients. The pathway most anticorrelated to risk is *Regulation of Autophagy* (*R* = −0.45, *p* = 1.1 × 10^–6^), which is consistent with the presence of *DRAM1* in Pr-61 and *DRAM2* in Pr-94. These genes are critical to apoptosis via induction of autophagy in the p53 tumor suppressor pathway^30^. The top three pathways most correlated to risk involve the chaperonin *TRiC/CCT* (*R* = 0.52, *p* = 2.0 × 10^–8^). This is consistent with the presence of *CCT3* and *CCT5* in Pr-72. *CCT3* is the component of *TRiC/CCT* responsible for regulating the function and levels of *STAT3*—a critical regulator in MM that facilitates evasion of apoptosis^31–33^. *CCT3* also regulates *CDC20*, which is required for cell cycle progression and modulates the antiapoptotic protein *MCL1*^34^. Moreover, *CCT3* and *MCL1* are among the genes whose expression is most strongly correlated to risk in t(4;14). Finally, gene set enrichment analysis on the differential expression of the extreme-risk Pr-68(+)/Pr-61(−) versus standard-risk Pr-68(+)/Pr-61(≥0) showed that the mitotic cell cycle (adjusted *p* = 3.5 × 10^–69^), DNA repair (adjusted *p* = 3.0 × 10^–23^), transcriptional regulation by *TP53* (adjusted *p* = 5.5 × 10^–15^), *MYC* activation pathway (adjusted *p* = 1.8 × 10^–9^), and glycolysis (adjusted *p* = 3.5 × 10^–9^) were among the most significantly overactive pathways in the extreme-risk subset.

**Fig. 4:**
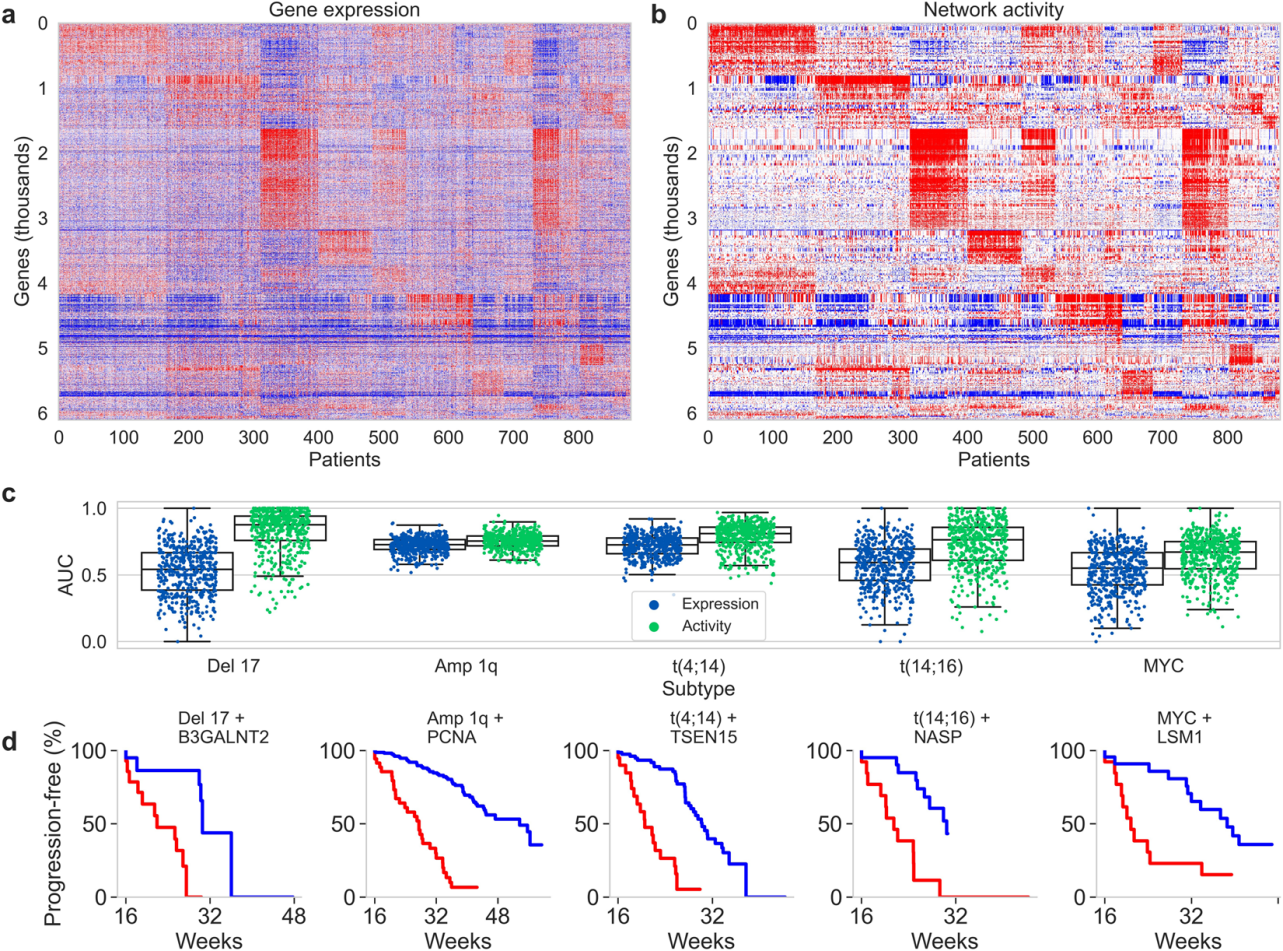
Network activity. **a**, **b** Heatmaps of (**a**) normalized gene expression and (**b**) network activity for all genes appearing in at least two regulons. **c** Boxplots comparing the prediction of high-risk subsets using normalized expression (blue) or network activity (green) of the genes as features. Each point overlaying the boxplots is a mean area under the ROC curve (AUC) from 5-fold cross-validation of the high-risk prediction. One hundred iterations were performed for each condition to avoid bias in the selection of training and test subsets. Center line, median; box limits, upper and lower quartiles; whiskers, 1.5× interquartile range. **d** Kaplan–Meier curves demonstrating the sub-stratification of clinical subtypes based upon the network activity of the most predictive individual or pair of genes as determined by the predictions in (**c**).

As a final test of the information contained in the CM TRN, we transformed the gene expression data to network activity (i.e., network-constrained gene activity) by applying a correction to the measured expression value of a gene based on the expression levels of the other genes to which it is mechanistically connected in the CM TRN. Figure 4a, b shows that the network activity preserves the large-scale patterns present in the gene expression data and appears much less noisy. We compared the predictive performance of a gene’s expression to its network activity in the high-risk clinical subtypes to test whether the network correction improved predictive power (see “Methods”). For all high-risk subtypes, the network activity outperformed gene expression (Fig. 4c). The sub-stratification of progression-free survival enabled by the network activity of single genes is shown by subtype in Fig. 4d. Finally, the genes that were most predictive of risk in MMRF IA12 across all subtypes were evaluated in the GSE24080 and GSE19784 test sets. In both cases, the network activity outperformed the gene expression for predicting risk (Supplementary Fig. 16). The ability of the CM TRN to improve the predictive performance of individual genes and apparently filter noise from the corresponding expression data are strong indicators that the relationships in the inferred network reflect meaningful biological mechanisms.

### *CRBN* activity is linked to high-risk genetic program Pr-68 through *CCNDBP1*

Activation of *E2F1* and *FOXM1*, and thus the genes of Pr-68, is known to occur via the *CCND1–CDK4* complex^35–37^. The cyclin D-binding protein *CCNDBP1* has previously been shown to interfere with the *CCND1–CDK4* complex, providing a putative mechanism to prevent Pr-68 activation and thereby halt the G1/S transition of the cell cycle^38,39^. We found that *CCNDBP1* is strongly deactivated in canonical MM translocation subtypes relative to other subtypes (*p* = 1 × 10^–73^ via Wilcoxon’s rank-sum test). This suggests that the translocations confer greater dysregulation of *CCND1*, facilitating constitutive activation of the cell cycle. Moreover, the TFs that drive the activation of *CCNDBP1* in the CM TRN are directly linked to the activation of the IMiD substrate *CRBN* (Supplementary Fig. 17), such that dysregulating the *CCND1–CDK4* complex may have the indirect consequence of decreasing IMiD sensitivity (see Supplementary information)^40^.

### Drug targets show subtype-specific risk stratification

**Fig. 5:**
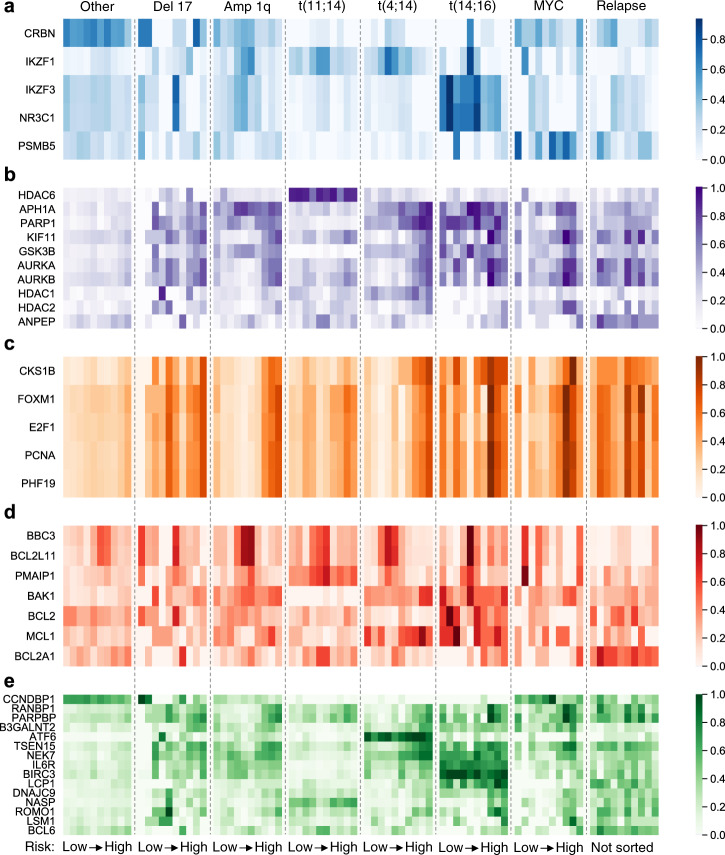
Network activity of drug targets across subtypes. **a** Targets of established baseline therapies. IMiDs: *CRBN*, *IKZF1*, *IKZF3*; dexamethasone: *NR3C1*; bortezomib and carfilzomib: *PSMB5*. **b** Targets of therapies in clinical trials for relapse-refractory MM (RRMM). **c** Genes associated with proliferation. **d** Genes directly involved in the intrinsic apoptosis pathway demonstrate subtype-specific differences in network activity. **e** Risk-associated and proposed target genes. These genes either classify a subtype via their uniformly high expression or are selectively active in the low- or high-risk subset of a clinical subtype.

We searched for subtype-specific relationships to the risk of disease progression in both the network activity and standard gene expression of genes whose corresponding protein is targeted by therapies available for MM. We considered both network activity and gene expression because they have complementary strengths. In theory, a gene that is well connected in the network will benefit from this correction, but a gene that is poorly connected (e.g., present in only 1 regulon) may be overcorrected due to a lack of information, such that the measured gene expression is more reliable. The correlation between risk of disease progression to network activity and gene expression is provided for each drug target by subtype in Supplementary Tables 1 and 2. The network activity of these important targets is visualized by risk decile for each subtype in Fig. 5. Subtype-specific relationships between the risk of disease progression and the network activity of various drug targets can clearly be seen and may be predictive of response to therapy. Specifically, some drug targets are highly correlated to the risk within a subtype (e.g., *AURKA/B* in Amp 1q, *PARP1* in t(4;14), etc.) and others exhibit high subtype-specific activity (e.g., *CRBN* in non-translocation patients, *HDAC6* in t(11;14), etc.). These relationships are quantified and discussed in detail in the Supplementary information. We note also that alternative methods to map drugs to subtypes may prove even more fruitful. For example, MINER enables gene set enrichment analysis of subtype-specific differentially expressed genes with respect to reference drug target databases (Supplementary Fig. 18).

### Relapse is characterized by the differential activity of five genetic programs

**Fig. 6:**
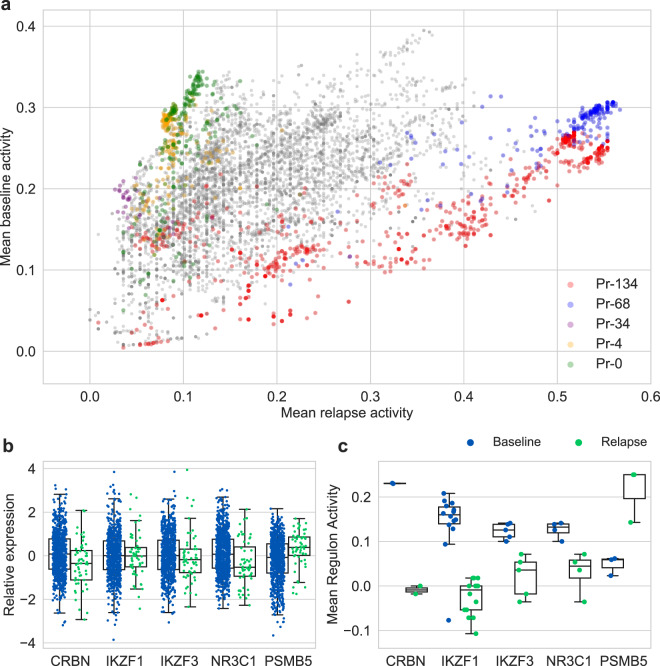
Network activity of genes at baseline versus relapse. **a** Each point depicts the mean network activity of a gene at relapse versus baseline. The genes of programs Pr-134, Pr-68, Pr-34, Pr-4, and Pr-0 are highlighted. **b**, **c** Boxplots of (**b**) gene expression and (**c**) mean regulon activity at baseline and relapse for target genes of baseline therapy (IMiDs: *CRBN*, *IKZF1*, *IKZF3*; dexamethasone: *NR3C1*; carfilzomib: *PSMB5*). Center line, median; box limits, upper and lower quartiles; whiskers, 1.5× interquartile range.

The genes with the greatest differences in network activity between baseline and relapse largely fall into five programs: Pr-0, Pr-4, Pr-34, Pr-68, and Pr-134 (Fig. 6). The program with the most extreme deactivation at relapse is Pr-34, which notably contains the genes *IKZF1* (IMiD target) and *PSMB7* (bortezomib target). The differential activity of regulons containing targets of baseline therapies were all decreased, whereas the regulon containing the carfilzomib target *PSMB5* was increased at baseline, suggesting plausible mechanisms for therapy escape and sensitization to new therapies at relapse. Notably, these patterns were not discernible through simple differential gene expression analysis. Program Pr-0 is also strongly deactivated at relapse, and notably contains several proapoptotic genes: *BCL2L11* (*BIM*), *BBC3* (*PUMA*), *BCL7B*, *TP53BP2*, and *TP53INP2*. On the other hand, programs Pr-68 and Pr-134 are the most strongly activated at relapse. Pr-68 is the aforementioned genetic program driven by *FOXM1* that characterizes high risk at baseline and is enriched with markers of proliferation. Interestingly, Pr-134 is not associated with risk at baseline (Cox HR = 1.0, *p* > 0.31).

### The genetic program with the highest activity at relapse reflects the microenvironment

**Fig. 7:**
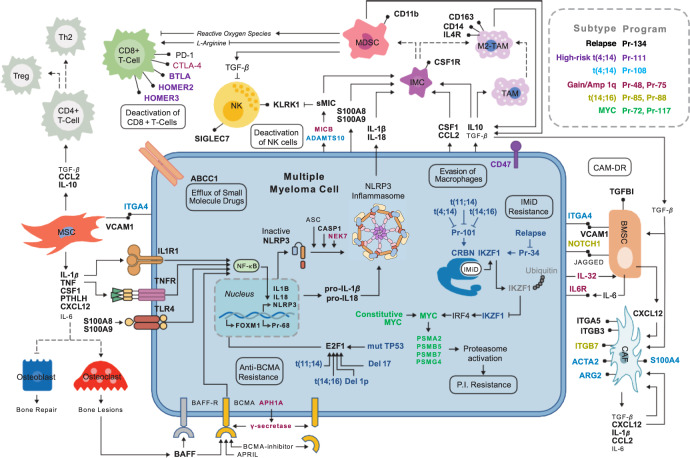
Pr-134 schematic. The genes of Pr-134 include markers of cell types characteristic to the bone marrow (BM) microenvironment, *NF-κB* signaling, and the *NLRP3* inflammasome. The high activity of these genes at relapse suggests the engagement of the BM microenvironment to facilitate escape from therapy and evasion of immune cell-mediated killing. The high-risk clinical subtypes are complementary to Pr-134 in escaping therapy and immune surveillance and they promote the proliferation of multiple myeloma cells by activating Pr-68 via *E2F1* and *FOXM1*. Bold gene names indicate that the gene is present in the program of the corresponding color (see legend).

Whereas Pr-68 reflects mechanisms of proliferation, Pr-134 comprises many markers of the immune-suppressed microenvironment. Especially noteworthy are signatures of myeloid-derived suppressor cells (MDSCs). In addition to the characteristic surface marker *CD11b*, many of the cytokines that promote the recruitment or generation of MDSCs (e.g., *M-CSF, G-CSF, IL-18, IL-1B, IL-10, CCL2, S100A8, S100A9, PTGER4*)^41^ are present in Pr-134. These cytokines, and those produced by the MDSCs themselves, are known to promote an immunosuppressive microenvironment. Signatures of BM stromal cells (BMSCs), M2-polarized macrophages, mesenchymal stem cells, osteoclasts, noncytotoxic T cells, anergic exhausted cytotoxic T cells, natural killer (NK) cells, and cancer-associated fibroblasts are also present in Pr-134 (Fig. 7). Finally, we note that gene set enrichment scores of *PD-1 Signaling* were significantly increased at relapse with respect to baseline (*p* = 3.5 × 10^–7^), suggesting a possible susceptibility to *PD-1* or *PD-L1* inhibitors.

## Discussion

MM presents heterogeneity of cytogenetics, mutations, gene expression, and clinical outcomes. Great strides have been made to characterize and understand these features, but we still lack a comprehensive map of the mechanistic links between them. Such a map will elucidate the genetic programs that are dysregulated in each subtype and provide points of actionable intervention in their mechanisms. We developed MINER to uncover the structure and hierarchy of patterns in gene expression from a mechanistic and causal perspective in MM, such that cytogenetics and mutations could be linked to their downstream effects on transcriptional regulators and genetic program expression. The profiles of genetic programs that are overexpressed or underexpressed in each patient constitute robust molecular signatures of dysregulation. By relating these signatures to their occurrence in each subtype of MM and their relationship to clinical outcomes, we developed a map relating the major features of myeloma heterogeneity by mechanisms that may be susceptible to currently approved or novel therapeutic interventions. Moreover, we have built an interactive web portal to facilitate a thorough but intuitive investigation of the CM TRN.

Despite the myriad combinations of mutations and chromosomal aberrations observed in MM patients, the transcriptional data are relatively well structured into 25 transcriptional states with 141 programs that can be further divided into 3203 regulons. This highlights a critical opportunity in the systems biology of cancer and the development of precision medicine: The effect of the myriad possible combinations of mutations is too great to study with statistical power in clinical analysis because specific combinations are too rare, but the end result is a discrete set of transcriptional profiles that *can* be studied effectively. Indeed, we see that transcriptional features such as genetic programs, regulons, network-constrained gene activity (i.e., network activity), and transcriptional states stratify the risk of disease progression better than mutations in the case of MM (Supplementary Fig. 9). Studying the transcriptional landscape of a disease in the context of a CM TRN enables relationships between the activities of genes and clinical outcomes to be traced to the putative causal effects of mutations. Moreover, those mutations that are known to predict risk can be better understood by investigating their downstream effects in the CM TRN.

We applied the CM TRN to investigate the influence of variable expression of drug targets, apoptosis regulators, and proliferation markers on the risk of disease progression. Individual genes (e.g., *CKS1B*, *PCNA*, *E2F1*, *FOXM1*, *PHF19*) and genetic programs (e.g., Pr-68) associated with cell proliferation were found to be the best overall predictors of risk. This agrees with previous studies but does not address the root cause of the cellular proliferation. However, we can use the CM TRN to see which mutations and regulators are upstream of these proliferative signatures in each clinical subtype of MM. We found that all high-risk clinical subtypes except t(4;14) are causally linked to the activation of Pr-68 by promoting activation of *FOXM1* via upregulation of *E2F1*. Neither expression nor network activity of *E2F1* was significantly upregulated in t(4;14), hence no causal link was established through *E2F1* downstream of t(4;14). We hypothesize that *FOXM1* is a master regulator of Pr-68 given that the genes in Pr-68 are highly enriched with confirmed ChIP-seq targets of *FOXM1* (*p* < 1.0 × 10^–55^). Activation of *E2F1* can occur as a direct consequence of dysregulating the G1/S cell cycle checkpoint via the common chromosomal aberrations observed in MM.

Although proliferation signatures are most predictive, the network activity of drug targets within the CM TRN also stratify risk at baseline. This is especially noteworthy in the case of the IMiD substrate *CRBN*, which is highly active in the subset of patients without clinical high-risk features. These patients do not exhibit strong proliferation signatures at baseline, so they likely benefit from low baseline aggressiveness of disease and high susceptibility to IMiD therapy. Subtype-specific differences in network activity suggest that complementary MM treatments can be particularly effective for specific subsets of MM patients. In particular, *HDAC6* inhibitors are rational therapies for t(11;14) patients, as they ubiquitously express *HDAC6*. Although experimental confirmation is required, this may signal a critical dependency of t(11;14)-driven MM on *HDAC6*. Moreover, the especially high activity of *PMAIP1* in t(11;14) supports the observed benefit of *BCL2* inhibitors in this subtype (Supplementary Fig. 19). Translocation t(4;14) also exhibits actionable subtype-specific risk signatures. The activity of *PARP1* and *TOP2A* each correlate with high risk in t(4;14) and enable proteasome resistance. Thus, *PARP* inhibitors and liposomal doxorubicin *(i.e.,* targeting *TOP2A*) are rational therapies for t(4;14) patients who become resistant to proteasome inhibitors. In addition, the correlation of *APH1A* activity to risk in t(4;14) and Amp 1q patients suggests that a gamma-secretase inhibitor may be particularly important for regimens involving monoclonal antibodies (mAbs). This agrees with the observation that gain of 1q21 confers poor prognosis in patients treated with Daratumamab (i.e., anti-*CD38* mAb)^42^. Finally, we note that virtually all subtypes exhibit a correlation between risk and the activity of the mitosis-related targets *AURKA*, *AURKB*, and *KIF11*, which may indicate that therapies against these targets will be effective in high-risk MM.

While the profile of apoptosis regulators was not strongly predictive of risk at baseline, significant changes were observed at relapse. Program Pr-0, which includes several proapoptotic regulators, shows much lower activity at relapse. Although *BCL2* and *MCL1* did not exhibit noteworthy changes at relapse, *BCL2A1* became ubiquitously active. Accordingly, we anticipate that *BCL2A1* may be an important therapeutic target in RRMM. Finally, evasion of autophagy-mediated apoptosis appears to be strongly predictive of risk in patients bearing translocation t(4;14). In particular, *DRAM1* and *DRAM2* become underexpressed in high-risk t(4;14) and *Regulation of Autophagy* pathway enrichments are strongly anticorrelated to risk.

A surprising result of this research was the observation of a strong signature of the immune microenvironment, despite the RNA-seq data being obtained from CD138+-purified MM cells. Markers of virtually all cells previously implicated in BM microenvironment-mediated resistance to therapy are present in program Pr-134, which becomes highly activated at relapse. This provides support to the hypothesis of a microenvironment-driven mechanism of resistance to therapy. The observation of these signatures in purified MM cells suggests that the mechanism of resistance may involve exosomes or other methods of delivering RNA from microenvironment cells. Although it is not yet clear which therapeutic strategies will be most effective for interfering with microenvironment-driven resistance, we note that targets related to nuclear factor-κB (*NF-κB*) signaling may be especially important. In particular, activation of *NF-κB* appears to be central to communication between the microenvironment and MM cells in the context of the CM TRN. *NF-κB* signaling is known to activate inflammasomes such as the *NLRP3* inflammasome, which generates *IL-18* and *IL-1B*^43^. These ligands stimulate cells of the microenvironment, such as MDSCs^44^, to ultimately produce ligands such as *S100A8*, *S100A9*, *TNF*, and *IL-1B*, all of which stimulate receptor-mediated *NF-κB* signaling and thus complete a circuit^41,43,45–48^. The genes of this circuit belong to Pr-134, suggesting that *NF-κB* signaling is a driver of microenvironment-induced resistance. Moreover, the *Inflammasomes* pathway shows significantly greater enrichment at relapse than baseline (*p* = 2.5 × 10^–6^).

We further analyzed the subtype of patients bearing translocation t(4;14) as an important use case for the application of the CM TRN. Some features are overexpressed across virtually all patients within this subtype and others are over- or underexpressed only in an extremely high-risk subset. Although it is not immediately clear which of these trends (i.e., ubiquitously overexpressed or risk-correlated) is more relevant for successful clinical intervention, they both provide valuable insights into the underlying biology of t(4;14) myeloma. For example, *HIF1A*, a critical regulator of angiogenesis and response to hypoxia, is overexpressed across virtually all t(4;14) patients. Interestingly, the distinct features of the extreme-risk group can largely be explained by the response to hypoxic stress and paracrine *IL-6* signaling with osteoclasts. In particular, the observed high expression of *IL-32* is known to occur under hypoxic stress by a *HIF1A*-dependent mechanism, leading to the secretion of *IL-32*, which promotes osteoclast differentiation and stimulates the production of *IL-6* in the microenvironment^49–51^. *IL-6* stimulates the *IL-6/JAK/STAT3* signaling pathway^33,52^, which is reflected in the high expression of *IL-6R* and *IL-6ST* in the extreme-risk Pr-68(+)/Pr-61(−) subset of t(4;14) patients. Although *JAK2* and *STAT3* expression is not elevated in the extreme-risk group, *CCT3*—a regulator of the function and levels of *STAT3*—is highly overexpressed and among the strongest correlates to risk across all t(4;14) patients. It has been reported that *STAT3* mediates escape from cytotoxic lymphocyte lysis under hypoxic conditions in a manner dependent on *HIF1A*, which indicates that *CCT3* may drive survival and is thus an important therapeutic target for high-risk t(4;14)^53,54^. Hypoxic stress also leads to the *HIF1A*-mediated activation of *ENO1* and subsequent increase in glycolysis, which is consistent with the significant overexpression of *ENO1* and functional enrichment of the glycolysis pathway in the extreme-risk subset^1^. In addition, hypoxia induces DNA damage, which could promote further activation of the DNA-repair program Pr-68. Indeed, the extreme-risk subset exhibits the highest levels of Pr-68 expression, which may reflect a synergistic relationship between the underexpression of Pr-61 and overexpression of programs Pr-68 and Pr-72.

Patients harboring t(4;14) demonstrate several additional signatures of interaction with the microenvironment. For example, program Pr-108, activated across all t(4;14) patients, contains *ITGA4*, which can directly engage BMSCs via *VCAM1*^55^. Moreover, 64% of t(4;14) patients overactivate program Pr-111, which contains the “don’t eat me” marker *CD47* that evades killing by macrophages^56,57^, and *BTLA*, *HOMER2*, and *HOMER3*, all of which suggest an escape from cytotoxic T cell killing^58,59^. The presence of both t(4;14) and Amp 1q shows synergistic activation of Pr-52 and Pr-75, such that patients harboring both abnormalities overactivate Pr-52 (*p* = 8.8 × 10^–9^) and Pr-75 (*p* = 7.7 × 10^–3^) relative to patients with either Amp 1q or t(4;14) alone. Programs Pr-52 and Pr-75 contain *ANXA2*, which directly promotes the differentiation of osteoclast progenitor cells into osteoclasts^60^, and contain *MICB*, which in soluble form has been shown to inactivate NK cells and cytotoxic T cells, steer macrophages to the tumor-promoting M2 phenotype, and stimulate the generation of MDSCs^61–64^. Lastly, Pr-75 contains *IL-6R* and *IL-32*, which promotes osteoclastogenesis and paracrine *IL-6* signaling as previously described^49,50^. Taken together, the “double-hit” combination of Amp 1q and t(4;14) bears signatures of a microenvironment that inactivates killing by cytotoxic lymphocytes, promotes the formation of immunosuppressive MDSCs and M2-polarized macrophages, and activates paracrine *IL-6* signaling. This suggests an immune-suppressive synergy as one element of the elevated risk of t(4;14)-Amp 1q “double-hit” patients.

The coherence of inferred programs across datasets, the strong performance of network activity-based predictions, and recapitulation of therapeutic escape mechanisms all provide support for the inferred network and methods of the analysis reported herein. We have demonstrated that it is possible to more accurately stratify patients into disease subtypes and predict risk based on dysfunctional genetic programs in a patient’s CD138+ myeloma cells relative to approaches based on just mutation or gene expression correlates. Because these genetic programs incorporate causation and mechanism, the network activity of known targets of Food and Drug Administration-approved therapies within these programs can be used as a means to prioritize therapy regimen for each patient. We envision that this approach will shortlist standard of care and investigational therapies, including therapies approved for other indications, that can be screened individually and in combinations in high-throughput drug screens using patient-derived CD138+ myeloma cells^65^. Thus, the MINER network can serve as the basis to accelerate the discovery of a personalized therapy regimen based on the unique dysfunction underlying a patient’s disease.

## Methods

### Data selection

In order to generate and test a MINER TRN of MM, we identified and preprocessed multiple publicly available datasets. Several gene expression datasets with associated clinical outcomes exist for MM, but the most extensive is that provided by the MMRF as a result of their CoMMpass study. In total, 1150 patients from 90 worldwide sites had BM samples analyzed every 6 months for 8 years. The samples were subject to many types of analysis, including genomic, cytogenetic, and transcriptomic analysis via RNA-seq of CD138+-purified MM cells. However, not all data from the CoMMpass study was publicly available at the time of this work. Nonetheless, the 881 samples with RNA-seq and translocation calls, 769 samples with matched clinical outcomes, and 734 samples with somatic mutation data available in the MMRF IA12 represent the richest MM dataset at the time of our analysis.

### Gene expression data processing

Gene expression data were downloaded from the IA12 data release of the MMRF. We analyzed the influence of the most highly expressed genes on all other gene values and concluded that special consideration was required to avoid batch effects resulting from highly expressed genes artificially lowering the TPM normalized expression values. In particular, the top 10 most highly expressed transcripts account for more of the mapped reads than the remaining 59,000+ transcripts combined, and the percent of mapped reads attributed to the top 10 genes is strongly anticorrelated to the number of unique transcripts detected (Supplementary Fig. 20). We implemented a custom normalization pipeline in Python that is similar to trimmed mean of *M* values (TMM)^66^ plus quantile normalization (see *miner.preprocess*: https://github.com/baliga-lab/miner3_mwall).

### Mutation data processing

Binary mutation matrices were generated such that columns were indexed by patient identifiers, rows were indexed by mutations, and the value of entry (*i*, *j*) = 1 if gene *i* was mutated in the patient sample *j* and (*i*, *j*) = 0 otherwise. The mutation calls used to populate this matrix were taken from the MMRF IA12 clinical data tables provided by the MMRF.

### Clinical data processing

Clinical outcomes data was downloaded from the MMRF Researcher Gateway. We used the GuanRank^67^ of time to progression-free survival and normalized the values to fall between 0 and 1. This processing optimizes the value of censored data for regression and classification problems. The code for this normalization is made available on our GitHub page (https://github.com/baliga-lab/miner3_mwall).

### Test dataset acquisition and processing

HOVON65 (GSE19784) and UAMS (GSE24080) datasets were downloaded from NCBI/GEO and processed with the oligo R package to provide RMA normalization. For gene-level files with multiple probes mapping to a single gene, log_2_ intensities were combined via the geometric mean. No quantile normalization or mean-variance scaling has been computed between studies. The gene expression data as provided was *Z*-scored and the normalized GuanRank was applied to the progression-free survival data.

### Network inference by MINER

MINER comprises many functions for the quality control processing, analysis, and predictive model generation from gene expression data in the context of an inferred TRN. The TRN is generated by a multistep pipeline that starts with unsupervised clustering of gene expression, then integrates prior knowledge databases (e.g., TFBSDB), and performs causal inference when the appropriate data (e.g., somatic mutations, copy-number variation, etc.) is available. The resulting MINER TRN is composed of units, called regulons, that comprise a set of coexpressed genes sharing a binding site for a regulator whose expression correlates to the first principal component (i.e., the eigengene) of the genes. Additional informations, such as upstream causal influences or risk of disease progression as a function of expression level, are associated with each regulon in the network to enable a modular structure. Tutorials of the MINER pipeline and all associated code is available on our GitHub page. The CM TRN presented herein was inferred using the MINER pipeline with the following parameter values: minimum number of genes in a coexpressed set of genes = 6, minimum number of genes in a regulon = 5, minimum magnitude of correlation between regulator and regulon eigengene = 0.2, maximum *p* value of binding site enrichment within coexpressed genes = 0.05.

### Calculation of regulon activity

For each normalized sample, the genes were ranked from lowest to highest expression and partitioned into three equal parts: a lower, middle, and top third. Given satisfactory normalization of the gene expression, e.g., by TMM normalization or the related method proposed in this work, we can form a null hypothesis that a random selection of genes with no coexpressed relationship will tend to distribute evenly between the top, middle, and bottom third of the ranked genes. We can then use a binomial distribution with *p* = 1/3 to model the probability that *k* genes fall into the same third given a selection of *N* genes, where *N* ≥ *k*. A default *p* value of 0.05 is used as a cutoff for rejecting the null hypothesis that the chosen set of genes are not coexpressed. Genes that pass this coherent cutoff in the lower third are labeled “underexpressed” and those that pass in the upper third all labeled “overexpressed”. All other cases are assigned a label of “neither.” Accordingly, we generate a matrix with values {−1, 0, 1} for the discrete activity of all regulons in all samples. When continuous values of regulon activity are necessary, we prefer to use the regulon eigengene—the first principal component of the regulon gene expression.

### Differential regulon expression analysis

A matrix of regulon eigengenes was created by computing the first principal component of the regulon gene expression for each regulon in the network. Differential regulon expression analysis proceeds by defining two groups, such as the patients of a transcriptional state and the complimentary group of all other patients not belonging to that state. For each regulon, a Wilcoxon’s rank-sum test is performed by comparing the eigengene expression in the two groups. A *p* value cutoff of 0.05 is used to determine significance.

### Generation of TF–TF network

Given a list of TFs, MINER infers a TF–TF network in three steps. First, Least Absolute Shrinkage and Selection Operator (LASSO) regression models are generated for each TF in the list to predict its expression using a subset of the other TFs in the list as predictors. Specifically, the TF list is a subset to include only TFs with a binding site for the target TF in TFBSDB or a CHiP-seq database. The second step is to prune the LASSO models to minimize the number of TF predictors necessary to maintain the same level of predictive accuracy. Finally, the LASSO coefficients of each predictor TF for each target TF are defined as weighted edges to connect the TFs in a network.

### Comparison of gene expression and network activity for subtype risk stratification

For each high-risk clinical subtype, we classified the 30% of patients who were highest risk by GuanRank^67^ as truly high risk and classified the other 70% as low risk. We then randomly split the samples into a training set and a test set (i.e., for each high-risk subtype), identified the gene that best stratified risk in the training set, and quantified its predictive performance in the test set by the area under the ROC curve (AUC). We repeated the prediction 100 times with random patient selections to generate a distribution of AUC scores.

### Inference of miRNA regulation

The effects of miRNA regulation were inferred by the Framework for Inference of Regulation by miRNAs (FIRM)^68^. The MINER pipeline can be directly applied to miRNA regulators in the same fashion as for TFs, but the lack of miRNA-seq data severely limited the reliable quantitative detection of miRNA transcripts. In this case, MINER defaults to testing for the enrichment of miRNA targets in coexpressed clusters, but does not enforce correlation of the miRNA expression. FIRM enables enrichment analysis to infer miRNA regulation when coexpressed gene sets are available, but reliable miRNA-seq data are not. We used the default parameters and significance thresholds of *p* = 0.05.

### Analysis of patients at first relapse

There are only 39 patients with baseline gene expression, first relapse gene expression, and clinical outcomes data in MMRF IA12. Thus, pairwise comparisons are feasible, but limited in sample size. Of these 39 patients, 21 had no translocations or high-risk features at baseline, 8 were t(11;14) subtype, 7 exhibited Amp 1q, 4 were t(4;14) subtype, 2 exhibited MYC overexpression, and 1 exhibited Del 17. Therefore, pairwise comparisons by subtype are statistically underpowered, with the possible exception of patients with no translocations or other high-risk features. Moreover, these 39 patients were significantly at higher risk (*p* < 3 × 10^–7^) than the remaining baseline patients who did not have matched relapse profiles in MMRF IA12, presumably because these patients relapsed faster and thus were the ones with data available. Given the limited relapse data available, we pooled all relapse samples (*N* = 56) with expression, even though 17 samples did not have matching baseline expression and clinical outcomes, and compared these profiles against the pool of all baseline expression profiles.

### Differential pathway analysis

A matrix of normalized pathway enrichments versus patient sample ID was generated by applying gene set enrichment analysis to each patient gene expression sample in MMRF IA12. Statistically significant differences in the pathway enrichments between two groups (e.g., baseline versus relapse) were calculated by the Wilcoxon’s rank-sum test.

### Risk prediction

We performed Ridge regression (scikit-learn) against the normalized GuanRank of PFS. The MMRF regulon activity was subset to include only the 20% of patients with the highest risk and 50% of patients with the lowest risk. We omitted moderate patients only when *training* the predictor. Many of the patients who we omitted during the model training had censored survival data, so they could have been anywhere from intermediate-risk to very low-risk. We excluded them during training to enable a comparison of patients who were clearly low- or high-risk. The reported performance metrics were evaluated on all patients of a dataset, so there is no risk of misevaluating due to omitting patients with moderate outcomes. The 20% of patients with the highest risk (i.e., GuanRank score) in each dataset were labeled as high-risk and all others were labeled as low-risk for calculation of AUCs. The regularization parameter was selected by randomly splitting the regulon activity into a training and test set, then training a Ridge model on the training set, and finally calculating the AUC of the test set prediction. This was repeated 500 times and the optimized regularization parameter was selected as that which maximized the mean AUC of the 500 tests.

### Validation of univariate risk prediction

The top 100 genes that stratified risk in MMRF IA12 via gene expression were intersected with the top 100 genes that stratified risk via network activity, yielding 39 genes. These 39 genes were evaluated by area under the receiver-operating characteristic (ROC) curve (AUC) using their gene expression or network activity values as predictors of risk in GSE24080 and GSE19784. Random permutations of network activity values were used as a reference for random prediction.

### Association of additional information to causal flows

For each causal flow, we integrated additional information by using a custom pipeline (miner_output_merge.py) that included the following processing steps*:* (i) For a given regulon in each causal flow, gene members were collected and queried against OpenTargets database (https://www.targetvalidation.org/) to collect all drugs associated with a given gene for MM (get_opentargets.py). (ii) Similarly, functional enrichment with Gene Ontology (GO) biological process terms (Benjamini–Hochberg-corrected *p* value ≤ 0.05) was performed for each regulon (GO_enrichment.R), followed by (iii) association with Hallmarks of Cancer by using semantic similarity (Lin semantic similarity score >0.4)^7,69,70^ (goSimHallmarksOfCancer.R). (iv) Putative miRNA regulators via the FIRM pipeline were also associated with each causal flow as described before.

### Reporting summary

Further information on research design is available in the Nature Research Reporting Summary linked to this article.

## Supplementary information

Supplementary Information

Reporting Summary

## Data Availability

The data that support the findings of this study are available from the Multiple Myeloma Research Foundation (MMRF), but restrictions apply to the availability of these data, which were used under license for the current study, and so are not publicly available. Data are, however, available from the authors upon reasonable request and with the permission of the MMRF. The validation datasets were processed according to the methods of Mason et al.^24^ from the MAQC-II (GSE24080) and HOVON65/GMMG-HD4 (GSE19784) studies as described in the multiple myeloma DREAM challenge.

## Code availability

The Python code used to perform this research and several tutorial notebooks can be accessed on GitHub: https://github.com/baliga-lab/miner3_mwall.
