# Supplementary material for: Genetic program activity delineates risk, relapse, and therapy responsiveness in multiple myeloma
Source: NPJ Precis Oncol. 2021 Jun 28;5:60. doi: 10.1038/s41698-021-00185-0 (PMC8239045; doi:10.1038/s41698-021-00185-0)
Supplement: Supplementary file 1 — Supplementary Information [file 41698_2021_185_MOESM1_ESM.pdf]

Supplementary Information for *Genetic program activity delineates risk, relapse, and therapy responsiveness in Multiple Myeloma*.

Supplementary Tables

Gene	Other	Del 17	Amp 1q	t(11;14)	t(4;14)	t(14;16)	MYC
CRBN	-0.12	--	-0.14	--	-0.16	--	--
IKZF1	--	--	--	--	-0.21	--	--
IKZF3	--	--	--	--	--	-0.38	--
NR3C1	--	--	-0.12	--	--	-0.40	--
PSMB5	-0.10	--	--	--	0.19	--	--
HDAC1	--	--	--	--	0.18	--	--
HDAC2	0.10	--	0.14	--	0.32	0.30	--
HDAC6	--	--	-0.16	--	-0.18	--	--
HDAC9	-0.10	--	--	--	--	--	--
APH1A	0.17	0.55	0.16	--	0.39	--	--
PARP1	0.14	0.36	0.15	--	0.44	--	--
KIF11	0.16	0.32	0.32	0.24	0.37	--	--
GSK3B	0.12	0.32	0.15	--	0.25	--	--
AURKA	0.13	--	0.24	0.17	0.43	--	--
AURKB	0.15	0.31	0.28	0.21	0.41	--	--
ANPEP	0.13	--	--	--	--	0.49	--

Supplementary Table 1. Significant correlations between risk of disease progression and network activity for targets of MM therapies. The spearman correlation coefficient is listed for all results satisfying $p < 0.1$.

Gene	Other	Del 17	Amp 1q	t(11;14)	t(4;14)	t(14;16)	MYC
CRBN	-0.10	--	-0.13	--	-0.31	--	--
IKZF1	--	--	--	--	--	--	--
IKZF3	--	--	--	--	--	--	--
NR3C1	-0.11	--	-0.12	--	-0.17	-0.56	--
PSMB5	--	--	--	--	--	--	--
HDAC1	--	--	--	--	0.17	--	--
HDAC2	--	--	0.12	--	0.27	0.31	--
HDAC6	--	--	--	--	--	--	--
HDAC9	--	--	-0.17	--	-0.25	--	-0.28
APH1A	0.09	0.31	--	0.17	0.24	--	0.29
PARP1	0.00	--	0.13	--	0.33	--	--
KIF11	0.13	0.35	0.25	0.24	0.38	0.32	0.33
GSK3B	--	--	--	--	--	--	--
AURKA	--	0.37	0.22	--	0.40	--	--
AURKB	0.12	0.34	0.28	0.27	0.36	--	--
ANPEP	0.14	--	--	--	--	--	--

Supplementary Table 2. Significant correlations between risk of disease progression and gene expression for targets of MM therapies. The spearman correlation coefficient is listed for all results satisfying $p < 0.1$.

Gene	Other	Del 17	Amp 1q	t(11;14)	t(4;14)	t(14;16)	MYC
CRBN	3.02E-02	3.92E-01	4.55E-02	2.09E-01	1.00E-01	7.59E-01	5.69E-01
IKZF1	4.36E-01	8.36E-01	2.42E-01	3.51E-01	3.48E-02	2.16E-01	8.54E-01
IKZF3	1.10E-01	7.11E-01	1.08E-01	8.88E-01	6.89E-01	2.08E-02	3.52E-01
NR3C1	1.60E-01	7.62E-01	9.50E-02	7.72E-01	7.87E-01	1.46E-02	5.79E-01
PSMB5	7.09E-02	8.95E-01	9.63E-01	6.46E-01	4.88E-02	4.11E-01	8.46E-01
HDAC1	7.72E-01	3.26E-01	3.24E-01	5.26E-01	7.21E-02	4.30E-01	7.92E-01
HDAC2	7.63E-02	1.69E-01	4.54E-02	2.47E-01	7.64E-04	6.74E-02	3.14E-01
HDAC6	9.50E-01	8.01E-01	2.39E-02	6.35E-01	6.21E-02	3.04E-01	5.60E-01
HDAC9	7.18E-02	3.90E-01	1.07E-01	4.33E-01	2.64E-01	5.30E-01	4.91E-01
APH1A	1.92E-03	6.40E-04	1.92E-02	8.53E-01	4.76E-05	5.73E-01	2.98E-01
PARP1	1.16E-02	3.36E-02	3.75E-02	3.22E-01	3.49E-06	8.45E-01	7.11E-02
KIF11	4.53E-03	6.36E-02	4.30E-06	2.69E-03	9.96E-05	2.14E-01	1.29E-01
GSK3B	2.70E-02	6.14E-02	3.14E-02	3.62E-01	8.77E-03	8.58E-01	2.50E-01
AURKA	1.81E-02	1.27E-01	4.58E-04	3.66E-02	5.61E-06	1.48E-01	2.02E-01
AURKB	5.56E-03	7.02E-02	3.85E-05	9.34E-03	1.57E-05	1.39E-01	2.04E-01
ANPEP	1.73E-02	9.03E-01	4.91E-01	5.28E-01	9.96E-01	2.30E-03	4.53E-01

Supplementary Table 3. Spearman correlation *p*-values of risk of disease progression versus network activity for targets of MM therapies. Risk of disease progression was quantified by the GuanRank algorithm.

Gene	Other	Del 17	Amp 1q	t(11;14)	t(4;14)	t(14;16)	MYC
CRBN	7.81E-02	4.49E-01	6.01E-02	1.28E-01	1.49E-03	3.86E-01	9.84E-01
IKZF1	2.45E-01	1.11E-01	8.77E-01	6.24E-01	8.66E-01	4.89E-01	5.58E-01
IKZF3	2.68E-01	3.50E-01	2.63E-01	8.57E-01	6.13E-01	6.89E-01	1.57E-01
NR3C1	4.80E-02	6.19E-01	8.37E-02	6.18E-01	7.86E-02	3.37E-04	1.67E-01
PSMB5	6.85E-01	3.09E-01	9.41E-01	8.98E-01	4.12E-01	8.76E-01	6.70E-01
HDAC1	4.08E-01	7.88E-01	3.66E-01	9.99E-01	7.47E-02	1.41E-01	6.51E-01
HDAC2	8.98E-01	3.12E-01	8.04E-02	3.69E-01	5.59E-03	6.20E-02	5.80E-01
HDAC6	8.15E-01	4.63E-01	9.48E-01	3.39E-01	6.60E-01	6.57E-01	2.51E-01
HDAC9	4.61E-01	4.40E-01	1.29E-02	6.84E-01	1.01E-02	1.80E-01	9.72E-02
APH1A	8.85E-02	6.99E-02	1.91E-01	3.89E-02	1.22E-02	2.78E-01	8.43E-02
PARP1	3.89E-01	4.72E-01	6.35E-02	7.90E-01	6.77E-04	6.17E-01	3.51E-01
KIF11	2.25E-02	3.72E-02	2.59E-04	2.76E-03	7.14E-05	5.53E-02	4.84E-02
GSK3B	2.42E-01	6.44E-01	7.14E-01	4.27E-01	2.11E-01	1.12E-01	6.48E-01
AURKA	2.07E-01	2.82E-02	1.69E-03	1.02E-01	2.13E-05	1.36E-01	1.13E-01
AURKB	3.35E-02	4.89E-02	4.23E-05	8.50E-04	1.54E-04	2.12E-01	1.22E-01
ANPEP	1.35E-02	1.76E-01	1.73E-01	6.45E-01	4.43E-01	5.18E-01	1.96E-01

Supplementary Table 4. Spearman correlation *p*-values of risk of disease progression versus gene expression for targets of MM therapies. Risk of disease progression was quantified by the GuanRank algorithm.

Supplementary Figures

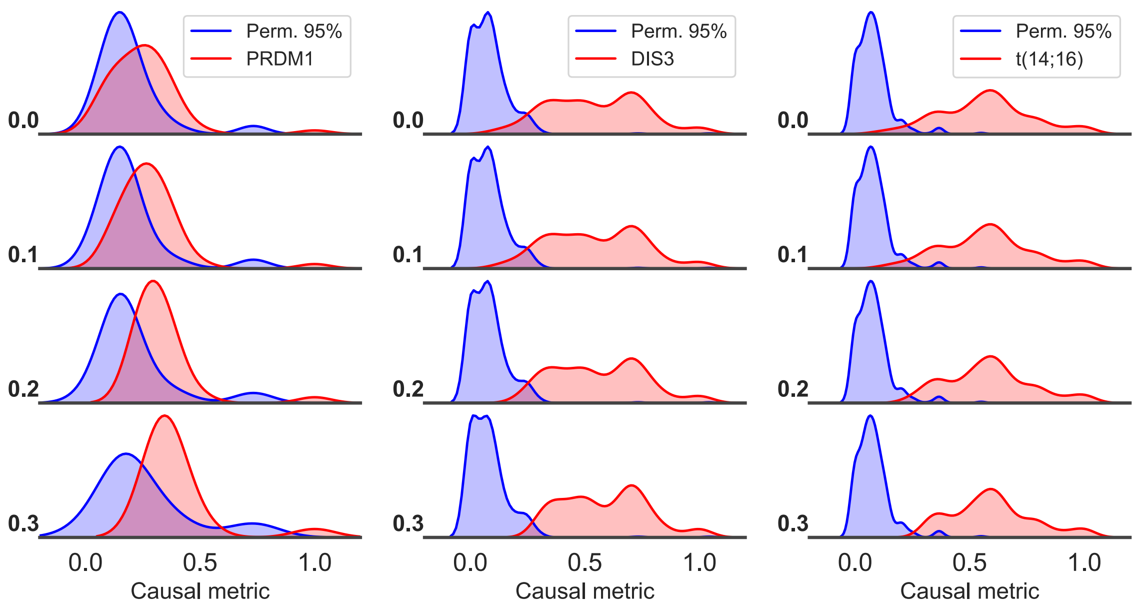

Supplementary Figure 1. Causal inference significance plots. Causal significance plots show how the observed distributions (red) of an important causal metric in the representative mutations of PRDM1 (left), DIS3 (middle), and translocation t(14;16) (right) compare to the distributions generated by random chance (*i.e.*, no causal effect; blue) for cutoff values of 0.0-0.3. The causal metric measures the flow through a regulator in the context of a mutation. Specifically, it is the fraction of regulons downstream of a regulator that are both differentially expressed and properly aligned (*e.g.*, activated or repressed) in the context of a mutation.

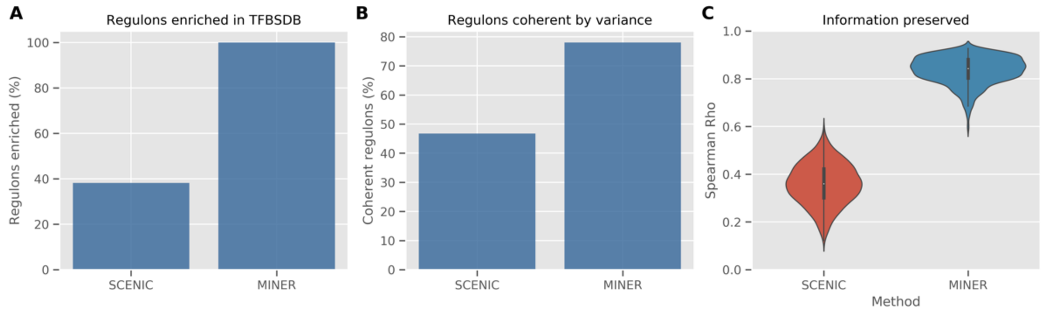

Supplementary Figure 2. Mechanistic inference benchmarking of MINER versus SCENIC. A, The percent of regulons enriched at a significance of $p<0.05$ with binding sites for the corresponding regulator in the transcription factor binding site database (TFBSDB) is 100% using MINER, but less than 40% using SCENIC. **B,** The percent of regulons with variance less than expected by chance at $p<0.05$ is much greater using MINER versus SCENIC. **C.** The Spearman rank correlation of pairwise sample distances calculated in the gene expression space versus the regulon activity space shows that mechanistic inference by MINER better preserves the information (*i.e.,* the topology) of the gene expression than does SCENIC.

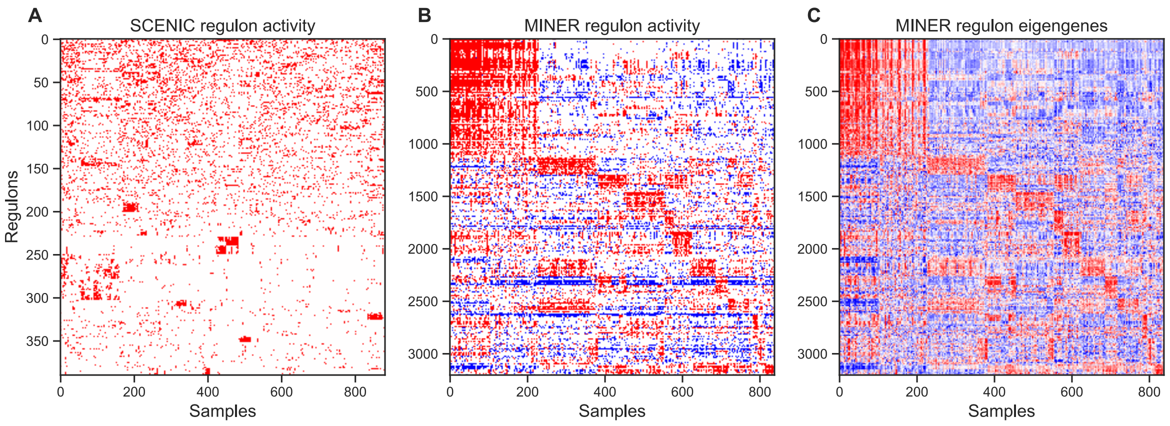

Supplementary Figure 3. Regulon activity comparison. A, Regulons discovered by SCENIC with activity calculated using the SCENIC AUCell and binarization method. **B,** Regulons discovered by MINER with activity calculated using the method proposed in this work. **C,** Regulons discovered by MINER with the first principal component (*i.e.*, the eigengene) value used as the network activity. Negative values are blue and positive values are red.

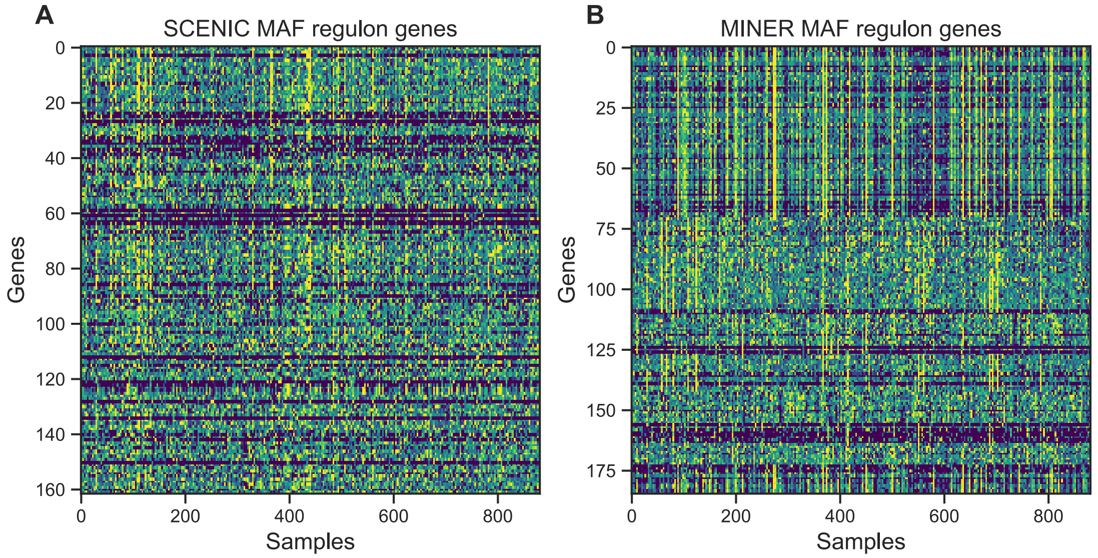

Supplementary Figure 4. Representative example of a regulon discovered by SCENIC versus MINER. A, The MAF regulon discovered by SCENIC features only genes that are predicted to be activated by MAF and shows a moderate degree of coherence in gene expression. **B,** MINER identifies several distinct regulons regulated by MAF, including genes that are activated (*e.g.,* rows 0-72) and repressed (*e.g.,* rows 73-110) by MAF, and the regulons tend to exhibit a higher degree of coherence.

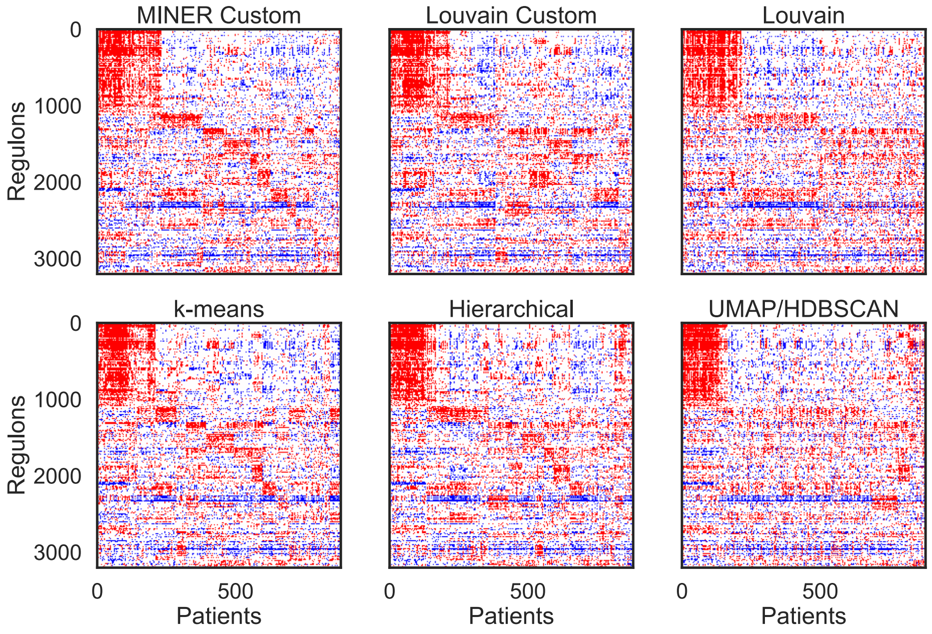

Supplementary Figure 5. Comparison of clustering methods for transcriptional state inference. Our preferred methods have finer resolution of state sizes. The custom method we introduce yields similar results to k-means and hierarchical clustering without requiring initial specification of the number of states.

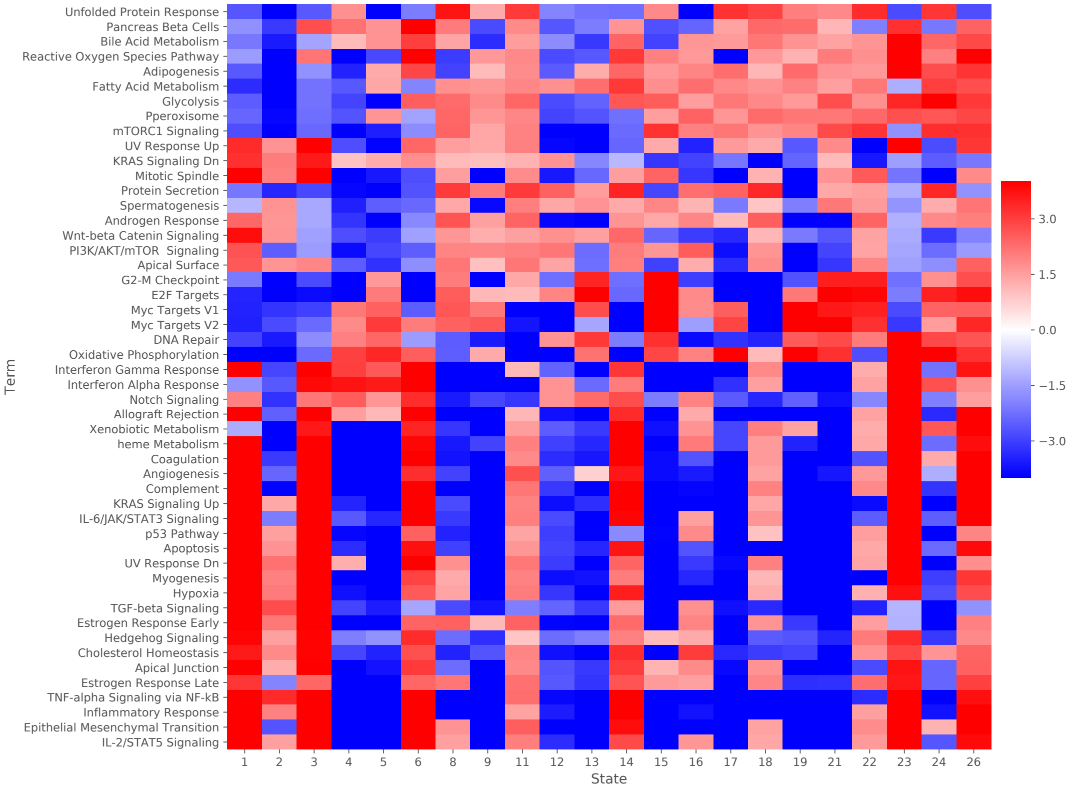

Supplementary Figure 6. Hallmark enrichments of transcriptional states. Heatmap of normalized enrichment scores from gene set enrichment analysis of the genes differentially expressed in each transcriptional state. The reference database was *MSigDb Hallmarks*.

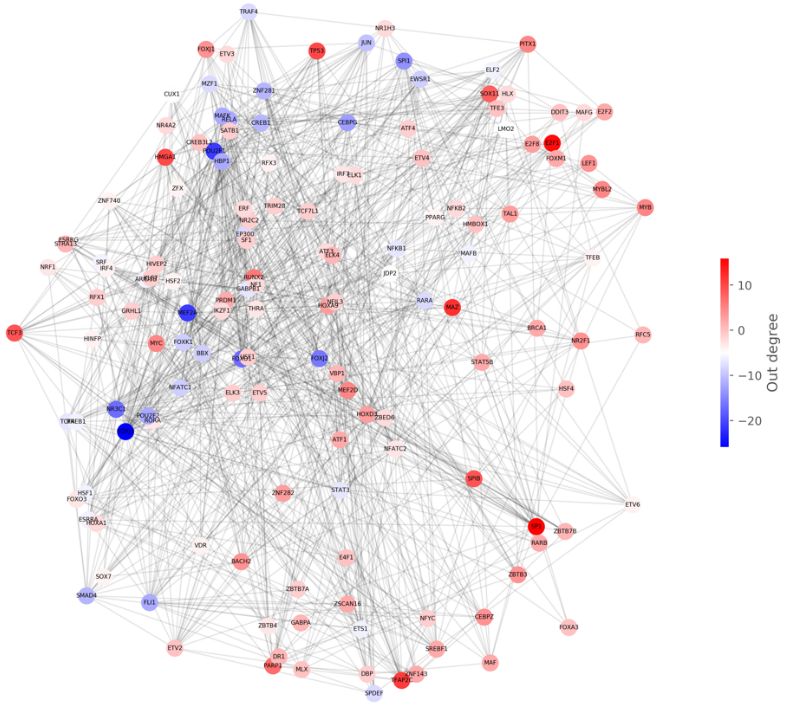

Supplementary Figure 7. Master regulator inference from TF-TF network. Master transcription factor (TF) network of transcriptional state 15 (*i.e.*, the highest-risk state). The color intensity of the TF node reflects the number of other TFs that are regulated by that TF (*i.e.*, the out degree). Red TFs activate the state, blue TFs repress the state.

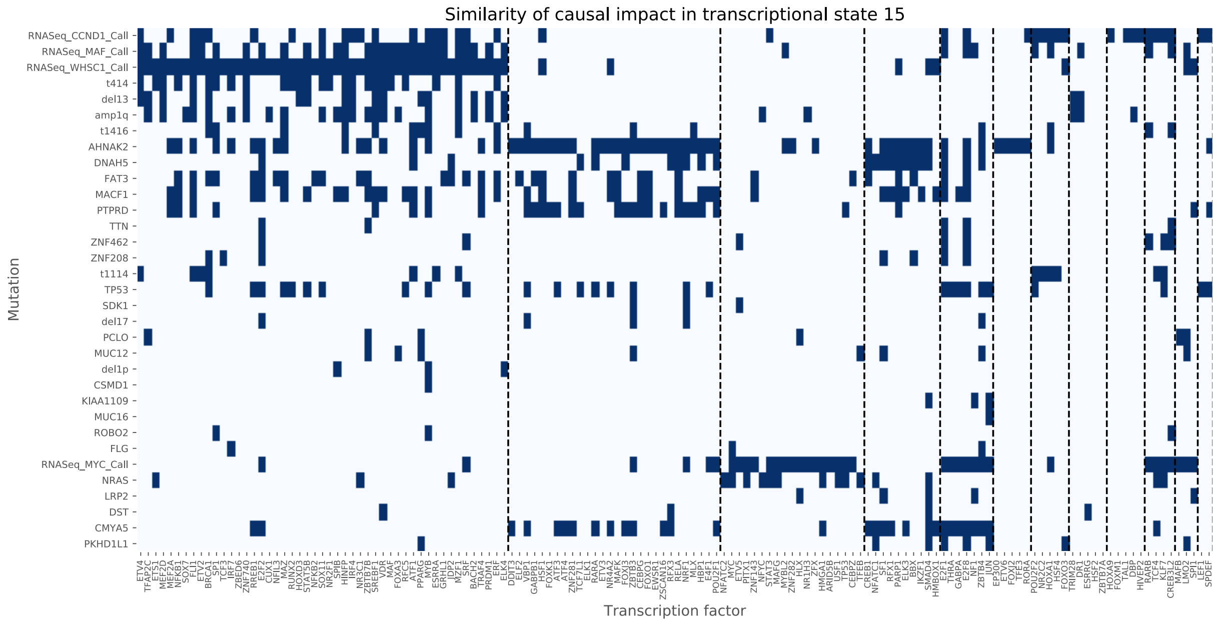

Figure S8. Different mutations activate specific subsets of a transcriptional state TF-TF network. Colored pixels indicate the causal activation of a transcription factor by a mutation. Dashed lines indicate borders between distinct subsets of TFs that tend to be activated by the same mutations. Mutations that the MMRF called according to RNA-Seq transcript levels are denoted by “RNASeq…Call”.

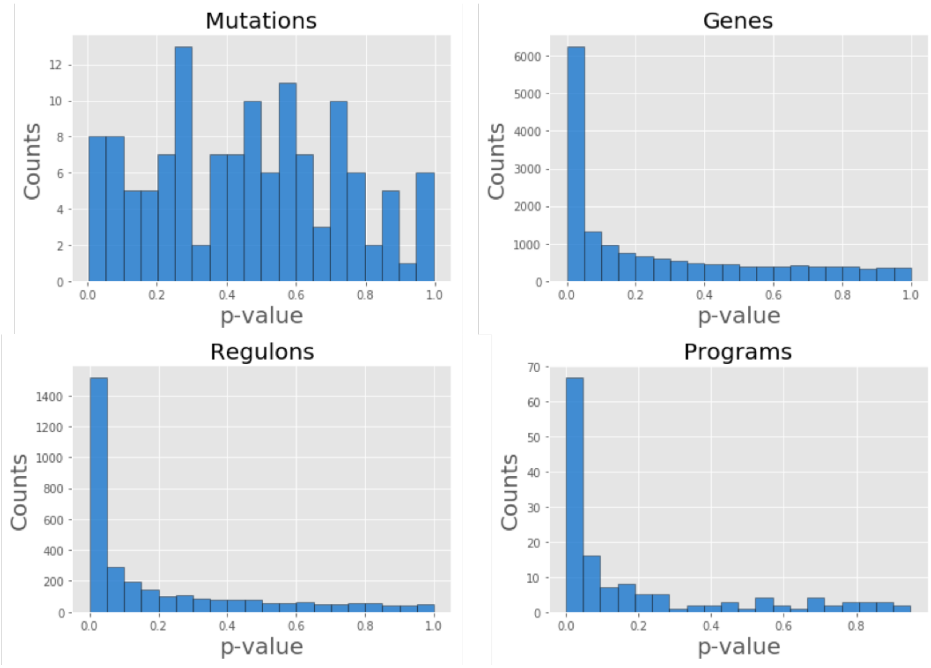

Supplementary Figure 9. Distribution of uncorrected p-values for various predictive features. The distribution of uncorrected p-values gives an indication of predictive power via setting expectations for false discovery rate. If the proportion of features with p<0.1 is significantly greater than 0.1, then the feature tends to stratify better than by random chance. Here we see that mutations do not stratify better than random chance, but that programs, regulons, and gene expression do.

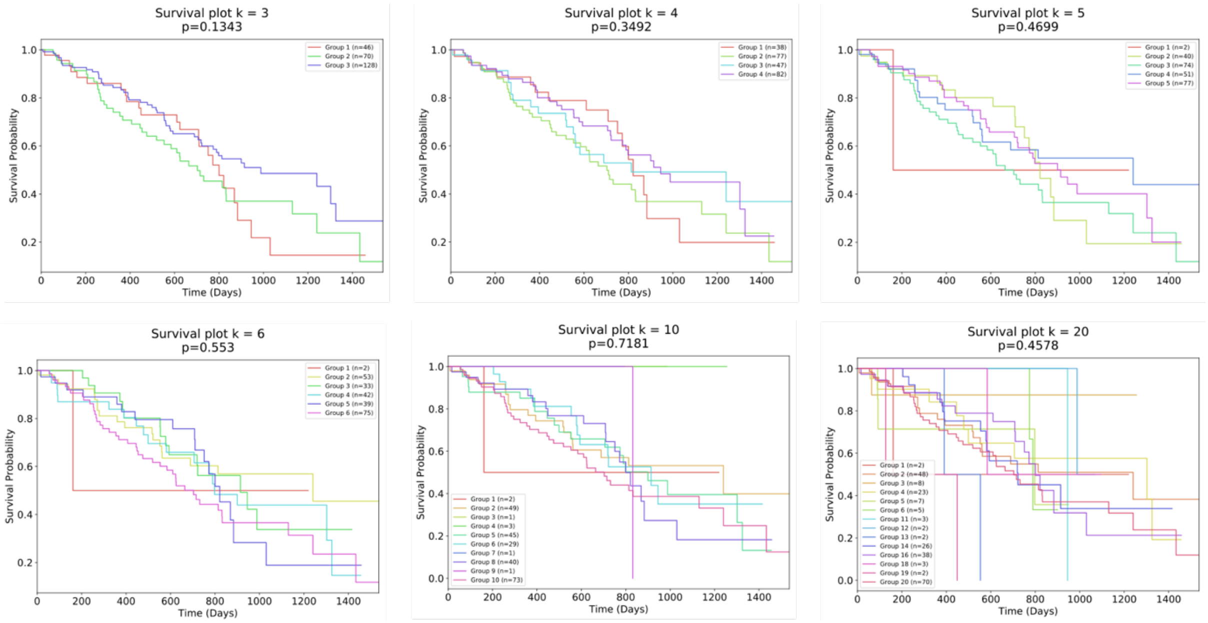

Supplementary Figure 10. Network-Based Stratification (NBS) does not stratify risk in MMRF IA12. We evaluated the risk stratification ability of the NBS method of Hofree *et al.*[1] using their default protein-protein interaction (PPI) network and the MMRF mutation data. The method did not significantly stratify the risk of patients, even when considering several different values for the number of subtypes (published default = 3).

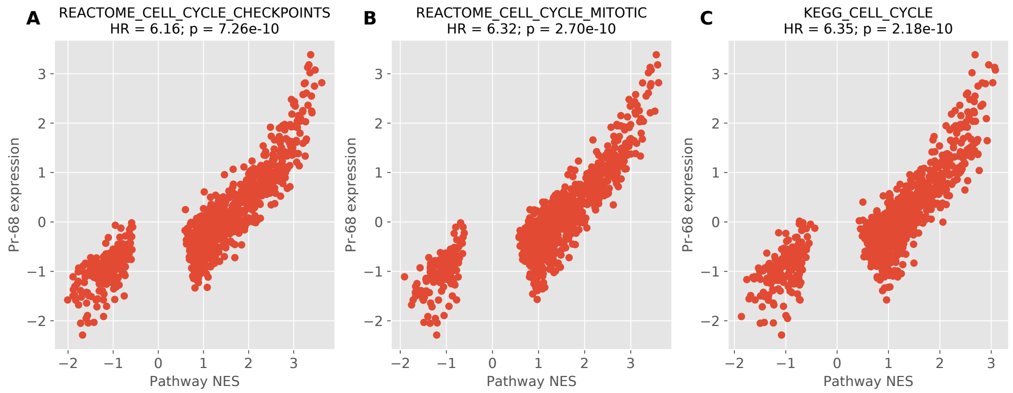

Supplementary Figure 11. Pr-68 expression versus cell cycle signatures. A-C, Scatterplots of program Pr-68 expression versus the three best-matched cell cycle signatures. Pr-68 expression was correlated to the normalized enrichment scores of the *MSigDb Pathways* database and the three most correlated cell cycle signatures were chosen. HR = Cox proportional hazards ratio.

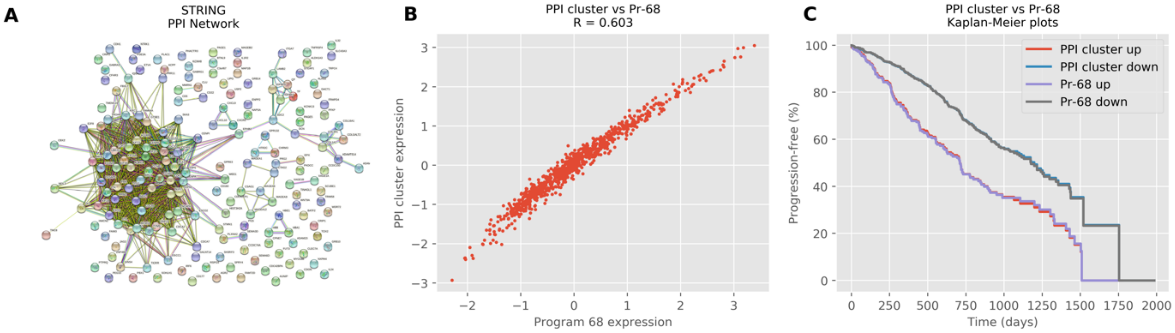

Supplementary Figure 12. Protein-protein interactions of differentially expressed genes reflect Pr-68. Differentially expressed genes (high- vs. low-risk) were analyzed with the STRING[2] online tool set to retain only high-confidence PPI as edges in the network following the method of Liu *et al.*[3] Three PPI clusters were identified using the online tool for clustering the PPI network (MCL). The primary cluster was very similar to program Pr-68 discovered by MINER.

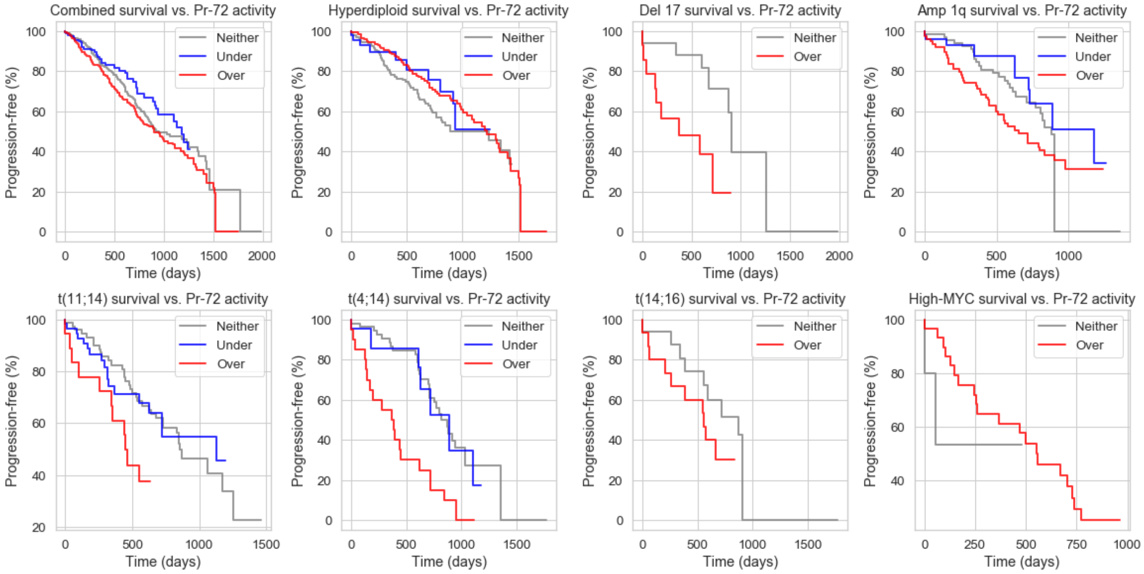

Supplementary Figure 13. Genetic program activity stratifies risk in subtype-specific manner. Over-expression of program Pr-72 increases risk in t(4;14) patients but not hyperdiploid patients.

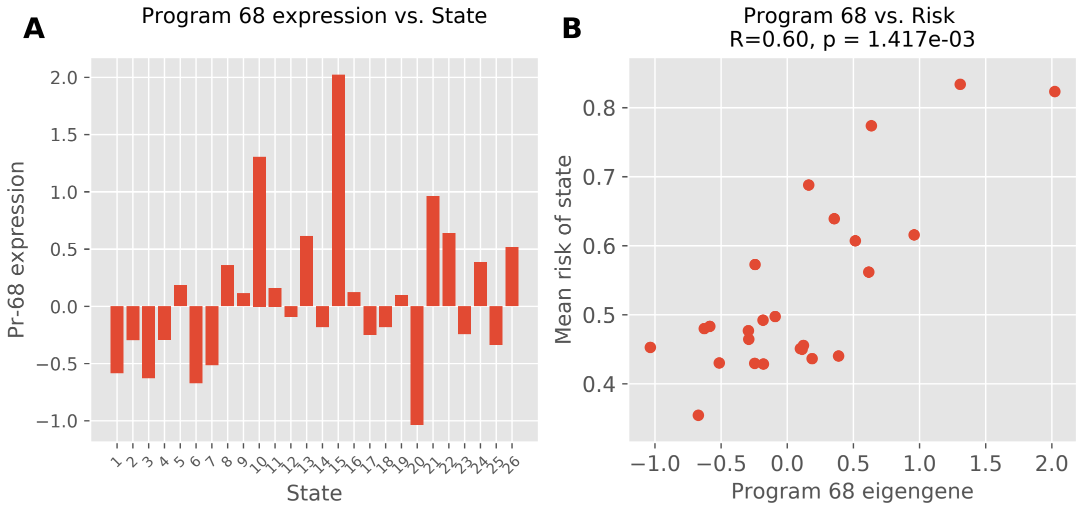

Supplementary Figure 14. Pr-68 expression versus state. A, The expression of program Pr-68, as quantified by the eigengene (*i.e.*, 1st principal component of program genes expression), varies across states. **B,** The Pr-68 eigengene correlates strongly to the mean risk of each state.

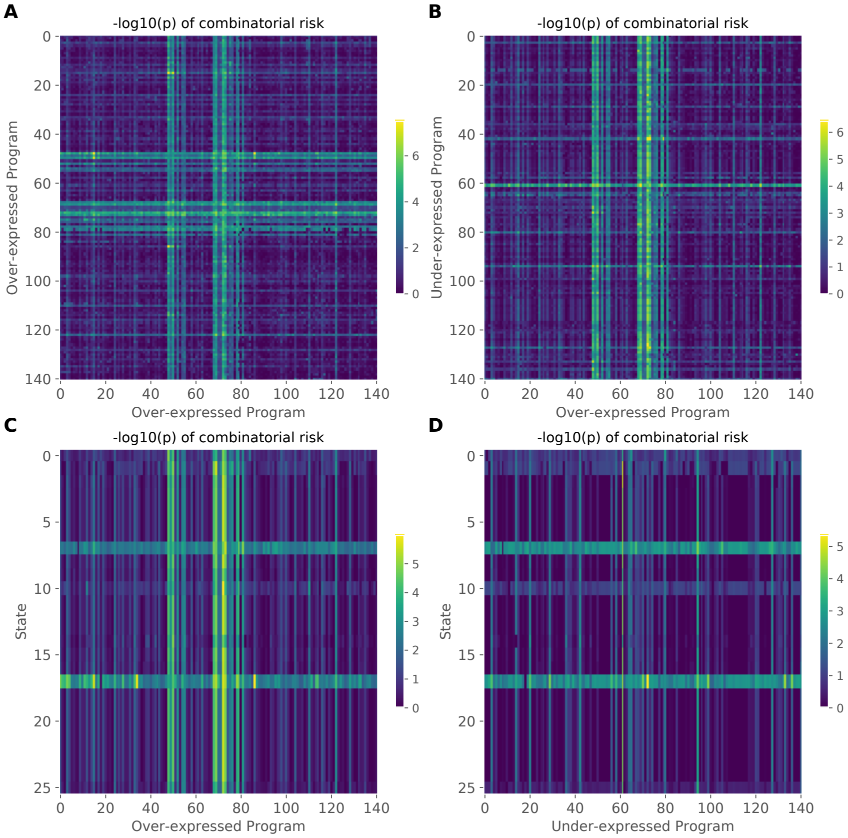

Supplementary Figure 15. Statistical framework for combinatorial risk. A-D, Kruskal-Wallis p-values for the pairwise-risk of combining (A) over-expressed programs, (B) over-expressed and under-expressed programs, (C) over-expressed programs and states, (D) under-expressed programs and states.

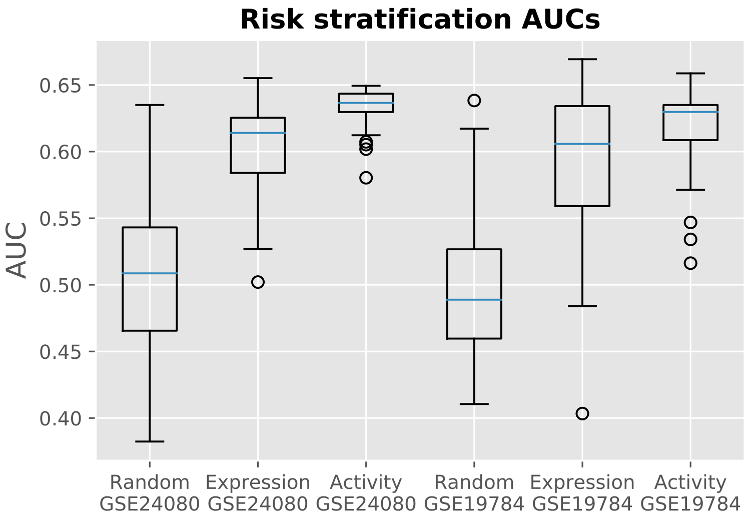

Supplementary Figure 16. Network activity improves univariate risk prediction. The individual genes most predictive of risk in MMRF IA12 were evaluated for their ability to stratify risk in the validation datasets (GSE24080 and GSE19784). In both cases the network activity outperformed the gene expression.

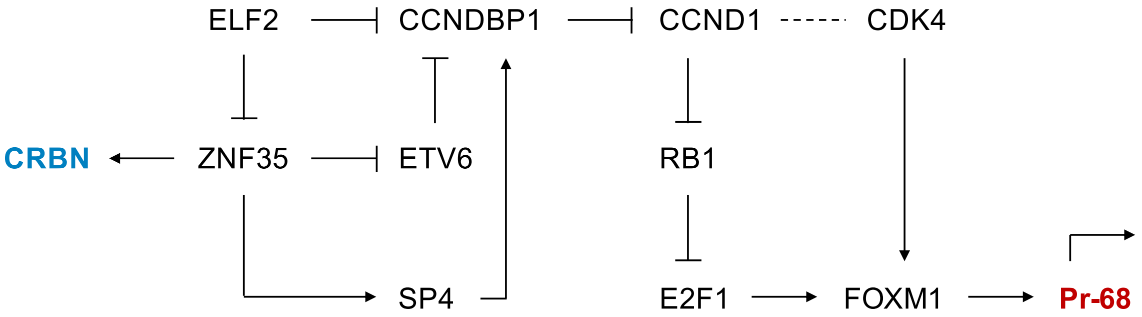

Supplementary Figure 17. Regulatory circuit connects CRBN and CCNDBP1 in CM TRN of MM. The activity of CRBN and CCNDBP1 are inferred to correlate due to shared upstream regulators of ELF2 and ZNF35. The link between CCNDBP1 and the CCND1-CDK4 complex connects this circuit to Pr-68.

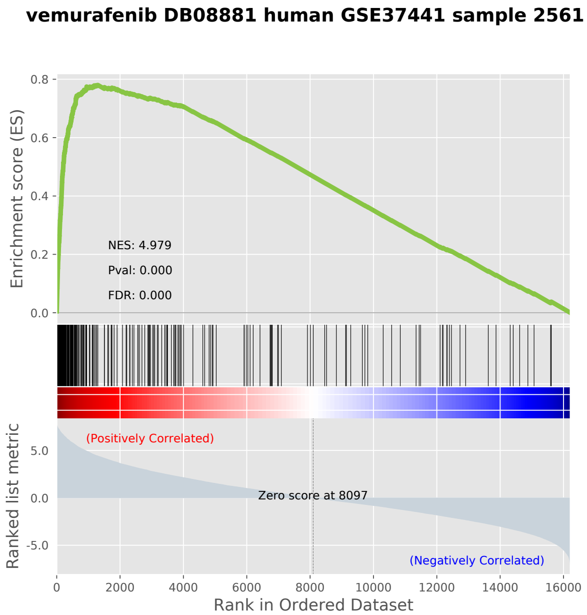

Supplementary Figure 18. Example application of a reference database to prioritize drug interventions. We compute the differential expression of genes in a state versus out of the state, then compare that to the *GEO drug perturbations down* database. The strongest hits represent drugs that would down-regulate (i.e., reverse) the over-expressed genes of the state. This approach can be used on any user-supplied differential expression data, for example from cell line experiments.

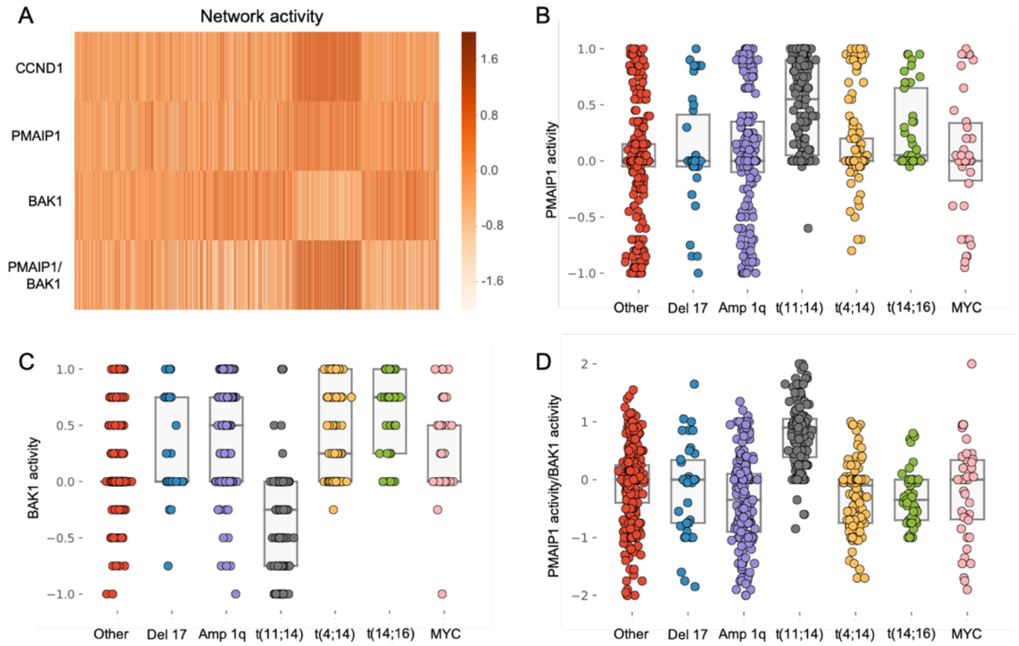

Supplementary Figure 19. Subtype-specific network activity profiles of BCL2-family intrinsic apoptosis genes. The network activity of genes in the BCL2 family that regulate intrinsic apoptosis signaling exhibit distinct profiles. **A,** The genes PMAIP1 and BAK1 are particularly noteworthy for their changes in activity upon high CCND1 expression (e.g., as occurs in t(11;14) patients). **B-D,** The network activity of (B) PMAIP1 and (C) BAK1 exhibit significantly different activity levels in t(11;14) patients in comparison to other subtypes, and (D) the ratio of PMAIP1/BAK1 is especially high in these patients.

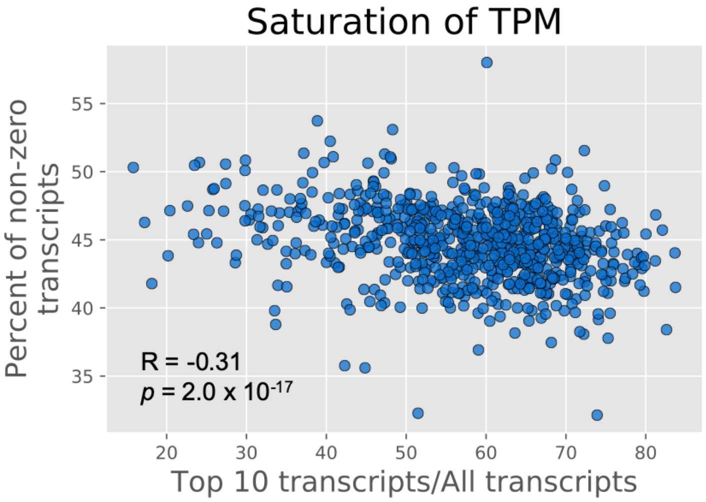

Supplementary Figure 20. Unwanted influence of highly-expressed genes on expression data. The percentage of transcripts that were detected (i.e., non-zero counts) via RNA-seq strongly anti-correlated with the percent of counts attributed to the 10 most highly expressed genes in any sample. This suggests that the highly-expressed genes are introducing bias into the experiments by saturating the reads and thus under-representing the expression of all other transcripts. This effect must be accounted for during pre-processing of the RNA-seq data by an approach such as TMM normalization that is not biased by outlier expression.

Supplementary Methods

Data normalization

Gene expression clustering in MINER requires scaled and centered gene expression data, as is commonly used in dimensionality reduction techniques like principal components analysis. While a standard Z-score transformation may be sufficient for pre-processing, we encourage the user to consider any experimental factors particular to their data set that might complicate the observed expression values. For example, in our multiple myeloma data set we noticed that some transcripts were expressed at such high levels it was reasonable to expect that other transcripts were effectively under-sampled due to competition with the abundant transcripts. In order to avoid this over-correction, *we generally recommend Trimmed means of M values (TMM) normalization*[4] *followed by Z-score transformation*. However, we tailored our normalization process to include additional steps that mitigate batch effects due to under-sampling of transcripts.

We start by transforming the counts data to transcripts per million (TPM) format. Then, for each sample, we calculate the median of the non-zero values and scale the expression so that the scaled non-zero median value is the same in all samples. We use non-zero values in order to prevent bias as a function of the number of non-zero values. Next, we quantile normalize the expression in each sample so that the distribution of gene expression values is the same in each sample, then we quantile normalize these values across samples (similar to a Z-score). This process is more aggressive than more common procedures like TMM normalization but offers greater mitigation of potential transcript under-sampling effects. This procedure is made available in MINER by the function “preProcessTPM”.

Supplementary Discussion

What do we mean by network inference?

The general definition of a *network* as a collection of nodes and the edges that relate them can represent many different things. When we refer to a network, we are generally speaking of causal mutations, regulators, and regulons as nodes. The causal mutations can have up-regulating or down-regulating edges directed to regulators, and the regulators can have activating or repressing edges to regulons. This complete collection of associations represents a global model (*i.e.*, one network for multiple myeloma), as opposed to a collection of local models (*e.g.*, t(4;14) network, t(11;14) network, etc.). However, we note that specific local networks can be obtained by pruning the global network to only contain regulons that are differentially expressed under a given condition, or by subsetting the global network to only those causal flows containing a specific mutation.

Network inference methods seek to discover a network of nodes (*e.g.,* regulators and target genes) and edges (*e.g.,* activates or represses) from a data set that can in turn be used to explain the processes that generated the observed data. In the case of transcriptional regulatory networks (also called gene regulatory networks), the goal of network inference is to connect regulators, like transcription factors, and target genes in the way that best explains the observed levels of mRNA in a gene expression data set. Several methods for transcriptional regulatory networks have been developed, such as GENIE3[5] (and the closely related GRNboost[6]), ARACNe[7], CLR[8], and cMonkey[9,10].

Mechanistic inference is a subset of network inference wherein biological evidence, such as the presence of transcription factor binding sites in the promoter regions of target genes, is used to increase the confidence of inferred regulatory relationships. Because additional information must be included, mechanistic inference generally involves a network inference algorithm and one or more input databases. In theory, any network inference algorithm could be combined with databases in this way, but in practice, there are few available methods that do.

How does MINER relate to other methods of network inference?

We developed MINER to provide a robust and efficient pipeline for mechanistic and causal inference (see definition of causal inference below) of regulatory networks that further incorporates tools for risk prediction and the discovery of patient subtypes. The closest alternative methods, to our knowledge, are SYstems Genetic Network AnaLysis (SYGNAL)[11] and Single-Cell rEgulatory Network Inference and Clustering (SCENIC)[6], which each provide some, but not all of the components available in MINER. SYGNAL was developed in our lab and served as a template for the pipeline design of MINER. The most important differences between them are that MINER uses mechanistic and causal inference algorithms that are much faster than those used in SYGNAL and that MINER provides many new tools for subtype discovery, regulon analysis, and risk prediction. Although SCENIC was originally developed for single-cell analysis, its algorithms also work for bulk RNA-seq data (e.g., its network inference algorithm was originally developed for bulk RNA-seq), and the mechanistic inference pipeline design is an even closer match to MINER than is SYGNAL. In particular, SCENIC identifies regulons and provides a measure of each regulon's activity in each sample. Because SCENIC is well-established and the closest available alternative to MINER, it is an ideal alternative method of network inference against which we can benchmark the performance of MINER.

Which algorithms does MINER use for mechanistic inference?

MINER splits its mechanistic inference module into three parts: 1) co-expression clustering, 2) enrichment analysis, and 3) subsetting of enriched co-expressed clusters into regulons. The co-expression clustering algorithm is novel but based upon intuitive principles. Our goal is to identify gene sets that are correlated and aligned (*i.e.*, concurrently over- or under-expressed) in the same patients. In order to find sets of correlated genes we begin by performing principal components (PC) analysis to find the dominant axes of covariance in the gene expression data. Genes whose correlation to a PC exceeds the 95th percentile are grouped to form an initial cluster of correlated genes. These groups of roughly correlated genes are partitioned into coherent subsets (*i.e.*, correlated and aligned) by identifying which genes are over- or under-expressed in the same patients. Once all of the correlated and aligned gene sets of are found, the clustering iteration stops and proceeds to the sample over-representation check. In this step, all of the correlated and aligned clusters are checked for over-expression in each patient. The 10% of patients with the greatest number of over-expressed clusters are removed from the expression data, along with all of the genes in the correlated and aligned clusters. This enables discovery of weaker signals in the expression data that had been dominated by the gene sets found in the previous iteration. We find that this is especially important when highly dissimilar patient subtypes are present. By iterating this procedure, signature patterns of a subtype are discovered and then the subtype is removed from consideration. A final clean-up step merges any clusters that have near-perfect correlation with each other.

The significance of the gene membership in each co-expression (*i.e.*, correlated and aligned) cluster is tested with respect to the target genes of the Transcription Factor Binding Site Database (TFBSDB). If the p-value of the hypergeometric test for the overlap between the co-expressed gene cluster and the target genes of a given regulator is less than p = 0.05, then all of the overlapping genes (*i.e.*, target genes of the regulator in the cluster) are selected and subjected to one final alignment step. The output of this step is a set of regulons – highly correlated and aligned genes with a shared TFBSDB binding site.

The robustness of any method that enforces transcription factor binding site information will be limited by the sensitivity and specificity of its reference database. For this reason, we previously developed and validated the TFBSDB[11]. In addition to the presence of TF binding site motifs, we required that DNase I hypersensitivity data from ENCODE[12] showed that the binding site overlapped with an open chromatin region and digital genetic footprint (DGF). This greatly reduced the amount of motif instances in the database that are unlikely to be bound by a transcription factor, as evidenced by ChIP-seq TF-target gene data. We note that 41 diverse cell and tissue types were used to generate the ENCODE data, so the accuracy of the predicted TF binding site information should not be strongly dependent upon cellular context. Nonetheless, it is important to acknowledge that the information contained in the TFBSDB, like any other reference database, is incomplete and may contain some erroneous TF-target gene associations.

How does causal inference in MINER compare to existing methods?

Causal inference of gene expression data is typically performed either by comparing the likelihood of different structural equation models (*e.g.,* NEO[13], FINDr[14]), evaluating the predictive performance of specific features in a machine learning model (*e.g.,* GENIE3[5]), or clustering mutations according to common occurrence in a pre-defined network (NBS[1]). In the case of GENIE3 (or the closely related GRNboost), the upstream causal anchors are derived from gene

expression, rather than mutation data, and although this is a subset of causal inference, we will discuss this separately as an instance of *mechanistic inference*.

The causal inference methods based upon structural equation model likelihoods compare the evidence that *mutation M* is upstream of *gene A* which is upstream of *gene B*, versus *gene B* being upstream of *gene A*, versus several other possible configurations of the relationships between these elements. While these methods are elegant and well-motivated in certain contexts, we feel that they are redundant in a pipeline that includes mechanistic inference. In particular, our mechanistic inference algorithm generates a network of relationships with regulators (*i.e.,* "*gene A*") upstream of several co-regulated genes (*i.e.*, "*gene B"*) including stringent tests for mechanistic evidence that goes beyond the likelihood ratios of various structural equation models (e.g., comparison to mechanistic databases with experimental evidence). Because we already have multiple lines of evidence that the regulators in our network are upstream of the target genes in their regulons, we do not require a separate causal inference method to compare the evidence of the orientation of the regulator and regulon genes.

The method of mapping related mutations identified by network-based stratification (NBS) to a network identified by a separate mechanistic inference algorithm represents an interesting and compelling approach to causal inference. The NBS method takes as inputs a network of gene-gene relationships and a mutation matrix (*i.e.*, mutation status of each gene in each sample), then applies non-negative matrix factorization to identify clusters within the network that are recurrently mutated in subsets of patient samples. In the original work, these patient clusters are used for risk stratification, but one could also use this as a causal inference method by defining the causal relationship *mutation M* activates/represses the genes of *subnetwork S*, in the context of *network N*. In this case, the *subnetwork S* is the cluster of related mutations to which *mutation M* belongs (*i.e.*, identified by the NBS algorithm), and the *network N* is the network provided as input to the NBS algorithm. Unfortunately, the restrictions of the NBS method (*e.g.*, the network must be acyclic) complicate the direct input of the network identified by the mechanistic inference module in MINER. However, we did attempt this approach using the network of related genes for use in cancer research (*i.e.*, the cancer subnetwork) provided by the creators of the NBS algorithm. Unfortunately, this method was not particularly useful for our purposes, as the patient clusters did not show significant risk stratification (**Fig. S10**), the subset of related mutations was not provided as an output, the coverage of genes in the cancer subnetwork was sparse, and the causal relationships did not easily fit into our desired *mutation M → regulator R → gene G* format. Nonetheless, we expect that a future iteration of this approach that resolves some of these challenges could prove to be a novel and compelling approach to causal inference.

The causal inference method presented in MINER considers several metrics to ask if the flow of activity through a regulator significantly changes in the context of a mutation. Specifically, for a given regulator, we look at all of its target regulons, which typically include both activating and repressing relationships, and we calculate how many of them are differentially expressed in the direction consistent with their regulator-regulon edge (*e.g.*, activated or repressed). We then compare the observed fraction of regulator-regulon edges that are differentially expressed and properly aligned to a null distribution generated by randomly assigning patients as having a mutation (*i.e.,* permutation analysis) (**Fig. S1**). Causal associations wherein the observed value is greater than the 95% confidence interval value from permutation sampling are called significant. A minimum acceptable threshold is set for this parameter to balance the number of causal flows (*i.e.*, Mutation → regulator → regulon) lost versus the significance of the ones retained. We found that the cutoff 0.2 was optimal.

Our approach additionally requires that the regulator be differentially expressed in the context of the mutation. Thus, a causal anchor is inferred above a regulator-regulon edge when the regulator and regulon are both differentially expressed, and a significant number (*i.e.*, from permutation analysis) of the other regulons downstream of the regulator are differentially expressed in the direction expected by the regulator-regulon edges. Moreover, the default in MINER is to use the network-constrained gene activity (*i.e.*, "network activity" as defined in the manuscript), rather than simply the gene expression, when calculating differential expression of the regulator. This provides a measure of flow to the regulator from its upstream nodes. Accordingly, an inferred causal flow in MINER requires contextual evidence for causal impact beyond the single mutation-regulator-regulon association being tested.

How does risk stratification in MINER compare to existing methods?

Risk prediction in precision oncology can take many forms, but typically relies upon mutation status, gene expression profiles, or clinical variables such as age and co-morbidity status. These variables are often combined or transformed in a process called feature engineering to improve their robustness, interpretability, or predictive power. The application of MINER to risk prediction centers on the generation of interpretable and predictive features (*i.e.*, feature engineering) and facilitation of their use as inputs in machine learning models. In this work, we are most interested in analyzing and interpreting the individual features of regulon activity, program activity, and transcriptional state membership, rather than proposing a single risk-predictive model (**Main text Figure 2A-E**). However, we do provide a regulon-based ridge regression machine learning model as a use-case of the features output by MINER. We note that this model does not include any clinical covariates such as age or sex, so the predictive power reflects the utility of the information contained in the regulons. We also provide an example of identifying and interpreting a subtype-specific risk model for patients harboring translocation t(4;14) (**Main text Figure 2F-G**), and a new statistical framework for calculating pairwise risk of features (**Fig. S15**).

The features generated by MINER enable risk prediction performance that is on par with the best available models in multiple myeloma (MM). The regulon-based ridge regression model yields area under the curve (AUC) values of 0.70 and 0.71 in the validation datasets (*i.e.*, datasets available in the literature that were not used to train the model). The recent crowdsourcing effort (Multiple Myeloma DREAM Challenge) to generate the best predictor of risk in MM found that bootstrapped integrated AUC values ranging from 0.65-0.70 characterized the best models.[15] The data sets used in that challenge included our validation data sets and a subset of the MMRF data set that we used to train our model, so it is reasonable to expect that the performance that we observe would translate to good performance in the challenge. However, the competition used private validation data and is now closed, so we cannot directly compare our model against the others. Nonetheless, it is clear that the regulon-based model demonstrates strong performance.

What is the significance of Pr-68 and how does it compare to alternative signatures?

Analysis of the features generated by MINER revealed that transcriptional program Pr-68 exhibits especially strong risk stratification. The observation that many of the Pr-68 genes are involved in cell-cycle processes raises the question: is the risk stratification exhibited by Pr-68 explained by its surrogacy for cell cycle progression? We have addressed this question by correlating the expression of Pr-68 to the normalized enrichment scores of all cell cycle pathways in the MSigDb database. Specifically, gene set enrichment analysis of each normalized patient expression profile resulted in a dataset of pathways versus samples that was correlated against Pr-68 expression. The three most correlated cell cycle signatures are shown in **Figure S11**. Interestingly, Cox proportional hazards regression shows that the expression of Pr-68 stratifies risk (HR = 8.8, p =

1.2 x 10^{-18}) much better than any of these cell cycle signatures (HR = 6.2, p = 7.3 x 10^{-10}; HR = 6.3, p = 2.7 x 10^{-10}; HR = 6.4, p = 2.2 x 10^{-10}).

We did identify one means by which the stratifying power of Pr-68 could be matched by a standard procedure. Differentially expressed genes were identified by comparing the 20% of highest-risk patients against the 50% of lowest risk patients in the MMRF IA12 normalized expression data set. The differentially expressed genes were analyzed with the STRING[2] online tool set to retain only high-confidence PPI as edges in the network following the method of Liu *et al*.[3] One large and two small PPI clusters were identified using the online tool for MCL clustering the PPI network (**Fig. S12A**). The primary cluster was very similar in composition to program Pr-68 discovered by MINER. Accordingly, the expression of the cluster correlated strongly to the expression of Pr-68 and had a similar Cox proportional hazards ratio (Pr-68: HR = 8.8, p = 1.2 x 10^{-18}; PPI cluster: HR = 8.2, p = 3.3 x 10^{-16}) (**Fig. S12B-C**). Although this approach was successful in identifying a high-risk feature, it required supervised information (*i.e.*, differential expression based upon risk labels) and generated little additional context or insights.

Given that the transcriptional states also strongly stratify risk, it is interesting to consider whether the expression of Pr-68 across states mirrors their relative risk. **Figure S14** shows that the expression level of Pr-68 varies greatly across states and that its average expression does correlate strongly to the average risk of patients in a state. This observation suggests that Pr-68 expression is an indicator of risk for progression regardless of the causal mechanisms driving disease in an individual because the different mechanisms characteristic to each state do not complicate its prognostic value. This is consistent with an interpretation of Pr-68 expression providing a surrogate of *in vivo* cell proliferation, since different biological mechanisms yield different rates of cell proliferation and these rates manifest in the different times to observed disease progression.

What drives the transcriptional states?

The similar transcriptional profiles shared by members of a state suggests a common set of causes that underly their observed gene expression. However, it is relatively unusual that patients in a state have more than one mutation in common. The root cause of specific expression patterns observed in each state may not be unequivocally provable without experimental validation, but the outputs of MINER provide sufficient information to generate strong, testable hypotheses.

At the simplest level, we can interpret the drivers of a state in terms of the transcription factors and epigenetic regulators that directly activate or repress its differentially expressed regulons and transcriptional programs. We can rank the importance of these regulators by how many of their regulons or target genes are differentially expressed in the state. The mutations inferred to be causally upstream of these regulators are potential causes of the state's gene expression. Although this simple approach provides a ranked list of potential causes, it does not shed light on the relationship between the differentially expressed regulons, and therefore may lack important systems-level information.

We can provide more context to the relative causal importance of regulators by inferring a TF-TF network that shows how regulators affect the activity of other regulators. MINER generates such a TF-TF network by a standard machine learning approach – LASSO regression – that accurately predicts the expression of each regulator as a function of a short list (*i.e.*, typically fewer than 10) of other regulators whose contributions are ranked by their model coefficient. When we do this for the regulators of all of the differentially expressed regulons in a transcriptional state, we see that there is a dense network of connections between the regulators (**Fig. S7**). In fact, a proof-of-

concept investigation of state 15 (*i.e.*, the highest-risk state) showed that any regulator in the TF-TF network could be reached by any other regulator within three degrees of separation (*i.e.*, two intermediate regulators). In this regard, the initial activation of entirely different parts of the TF-TF network will result in the entire TF-TF network becoming activated, thereby generating the same global pattern of gene expression. This is consistent with the observation that specific subsets of TFs tended to be activated by the same mutations (**Fig. S8**). Thus, mutations upstream of any of these regulators can, theoretically, activate the entire state. Despite the existence of many paths to activate the entire state, MINER does propose specific master regulators within the TF-TF network by identifying the regulators whose activation or repression is predicted to most quickly activate the entire network.

In addition to mutations, microenvironment features can be causes of specific gene expression profiles. MINER enables gene set enrichment analysis of the differentially expressed genes in each state with any reference database as a means to identify potential non-mutation causes of gene expression patterns. We provide the results of analyzing transcriptional state 15 with the MSigDb Hallmark pathways database as an example (**Fig. S6**). The hallmark targets of E2F and MYC are highly enriched in state 15. Separately, MINER identified E2F1 and MYC as potential master regulators of the state 15 TF-TF network. The convergence of these approaches on E2F1 and MYC suggest that their activation is an important aspect of achieving the gene expression profile characteristic of state 15. Moreover, MINER identified FOXM1 as a master regulator partner to E2F1 (*i.e.*, FOXM1 and E2F1 activate each other in the TF-TF network) and TP53 as a master regulator partner to MYC. Many mutations can cause the activation of these genes, which would consequently cause the observed differential expression of the regulons and programs characteristic to state 15.

Subtype-specific activity of drug targets

<u>*IMiDs – CRBN, IKZF1, IKZF3*</u>

All three IMiD targets[16] exhibited some degree of subtype-specific risk stratification. In addition to the significant difference in activity between translocation and non-translocation-bearing patients, CRBN sub-stratified risk in t(4;14) ($p_{expression}<1.5 \times 10^{-3}$, $p_{activity}<0.1$), Amp 1q ($p_{expression}<6.1 \times 10^{-2}$, $p_{activity}<4.6 \times 10^{-2}$), and patients without high-risk features or t(11;14) ($p_{expression}<7.9 \times 10^{-2}$, $p_{activity}<3.1 \times 10^{-2}$, labeled "Other" in **Tables S3-S4**). In each case, the CRBN activity was anticorrelated to risk, such that high activity of CRBN was favorable. This is in agreement with the role of CRBN in the mechanism of IMiD therapy, wherein it acts as a substrate for the degradation of IKZF1 and IKZF3. IKZF1 expression did not demonstrate significant correlation to risk in any of the subtypes, but IKZF1 network activity was significantly anticorrelated with risk in t(4;14) patients ($p_{activity}<3.5 \times 10^{-2}$). The low expression levels of IKZF1 and high coverage in the network (*i.e.,* member of 14 regulons) are precisely the conditions in which we would expect network activity to outperform standard gene expression. The higher activity of IKZF1 in low-risk t(4;14) patients is in agreement with its importance as a degradation target in the CRBN-dependent mechanism of IMiD therapy. IKZF3 gene expression was not correlated to risk in any subtypes, but IKZF3 network activity was anticorrelated to risk in t(14;16) patients ($p_{activity}<2.1 \times 10^{-2}$). This may indicate a therapeutic benefit to the IMiD-based degradation of IKZF3 in t(14;16) patients.

<u>*Dexamethasone – NR3C1*</u>

The gene expression and network activity of the dexamethasone target[17] *NR3C1* were both significantly anticorrelated to risk in t(14;16) patients ($p_{expression}<3.4 \times 10^{-4}$, $p_{activity}<1.5 \times 10^{-2}$). The

strong association between low levels of NR3C1 and high risk in t(14;16) indicate that escape from dexamethasone therapy is particularly important in this subtype. It is unclear, however, if the mechanism of escape involves down-regulation of NR3C1 within MM cells, or a smaller proportion of NR3C1-expressing cells (*e.g.*, in the microenvironment). The activity profile of NR3C1 is very similar to IKZF3, as these genes have similar connectivity in the CM TRN. Specifically, they are both strongly activated by ZEB1 and RORA. Thus, activation of these TFs may stabilize the high target activity of IKZF3 and NR3C1.

Bortezomib – PSMB5

The functional target of frontline proteasome inhibitors, PSMB5[18], shows relatively little subtype-specific correlation to risk at baseline. Although the gene expression of PSMB5 did not significantly correlate to risk in any subtype, its network activity was significantly correlated to risk in t(4;14) ($p_{activity}$<0.05). In particular, patients with low PSMB5 activity (*i.e.,* $PSMB5_{activity}<0$) tended to exhibit lower risk than patients with moderate to high activity ($p<7.1 \times 10^{-2}$).

HDACs – HDAC2 and HDAC6

HDAC expression profiles vary dramatically between subtypes. In particular, HDAC6 is ubiquitously expressed in t(11;14) patients, but not in other subtypes. Because HDAC6 is highly expressed across t(11;14) patients it does not correlate to their risk, however it may serve as a promising target in these patients if its expression reflects an essential function played in t(11;14) MM cells. Among the other HDACs, HDAC2 stood out for its subtype-specific correlation to risk, particularly in t(4;14) ($p_{expression}<5.6 \times 10^{-3}$, $p_{activity}<7.7 \times 10^{-4}$). HDAC2 also correlated to risk in Amp 1q ($p_{expression}<8.1 \times 10^{-2}$, $p_{activity}<4.6 \times 10^{-2}$) and t(14;16) ($p_{expression}<6.2 \times 10^{-2}$, $p_{activity}<6.8 \times 10^{-2}$) patients, though not as strongly.

Mitosis-related targets – AURKA, AURKB, KIF11

The mitosis-related targets AURKA, AURKB, and KIF11 are all members of Pr-68 and stratify risk well across several subtypes. AURKA, AURKB, and KIF11 are significantly correlated to risk in all subtypes other than t(14;16) and MYC (*i.e.*, patients with MYC overexpression). Although the network activity of KIF11 does not significantly correlated to risk in the t(14;16) and MYC subtypes, its gene expression does. The most significant risk stratification exhibited by these genes occurs in t(4;14) patients (AURKA: $p_{expression}<2.2 \times 10^{-5}$, $p_{activity}<5.7 \times 10^{-6}$, AURKB: $p_{expression}<1.6 \times 10^{-4}$, $p_{activity}<1.6 \times 10^{-5}$, KIF11: $p_{expression}<7.2 \times 10^{-5}$, $p_{activity}<1.0 \times 10^{-4}$)

PARP inhibitors – PARP1

The network activity of PARP1 correlated to risk in all subtypes other than t(11;14), t(14;16), and MYC. The correlation to risk was greatest in t(4;14) patients ($p_{expression}<6.8 \times 10^{-4}$, $p_{activity}<3.5 \times 10^{-6}$). We note that our CM TRN shows that PARP1 is an activator of TOP2A and NEK2. Inhibition of PARP1, TOP2A, or NEK2 have all independently been shown to overcome resistance to proteasome inhibitors.[19–21] The high activity of these genes in the highest-risk t(4;14) patients at baseline suggests that these patients would quickly become resistant to proteasome inhibitors, but would benefit from PARP1 inhibitors. We note that pegylated lysosomal doxorubicin targets TOP2A[22] and could also benefit high-risk t(4;14) patients.

Monoclonal antibodies – APH1A (gamma-secretase)

Monoclonal antibodies have emerged as promising next-generation therapies for MM but are susceptible to resistance by over-activation of the gamma-secretase protein complex, which cleaves the antibody targets from the cells. APH1A is an essential component of this complex and exhibits subtype-specific risk correlation. The APH1A gene is located on chromosome 1q and exhibits high network activity across the Amp 1q subtype, such that Amp 1q patients may be especially prone to gamma-secretase-mediated resistance to mAbs. Similar to PARP1, the network activity of APH1A correlated to risk in all subtypes other than t(11;14), t(14;16), and MYC. This motivates therapy by gamma-secretase inhibitors in these patients regardless of monoclonal antibody use, but especially in conjunction with them. We note that APH1A expression strongly correlates to PARP1 ($p<3.0 \times 10^{-31}$), TOP2A ($p<3.6 \times 10^{-6}$), and NEK2 ($p<6.0 \times 10^{-19}$), so it is unsurprising that the t(4;14) patients exhibit the strongest risk stratification by APH1A activity ($p_{expression}<1.2 \times 10^{-2}$, $p_{activity}<4.8 \times 10^{-5}$).

BCL2-family – BCL2, MCL1, NOXA, BAK1, BCL2A1

Figure 4D shows subtype-specific activity profiles of BCL2-related genes involved in the intrinsic apoptosis pathway. Patients harboring translocation t(11;14) exhibit a unique profile of these genes (**Fig. S6**), with high PMAIP1 (also called NOXA) and low BAK1 activity, such that these patients can be classified by their high PMAIP1/BAK1 ratio. It has been reported that the ability of PMAIP1 to bind the anti-apoptotic protein MCL-1 may underlie the success of BCL2-inhibitors in some patients.[23] Our network analysis supports that claim insofar as PMAIP1 shows much higher activity in t(11;14) than in other subtypes, and the responders to BCL2-inhibitors in MM have been significantly enriched with t(11;14) patients.[24] We note that the BCL2-family protein BCL2A1 is an interesting novel target for RRMM, as it becomes significantly over-expressed at relapse ($p<2.7 \times 10^{-5}$).

Melflufen – ANPEP

The Melphalan-flufenamide conjugate, Melflufen, targets MM cells that over-express the protein product of ANPEP and has shown clinical benefit in RRMM patients. We find that the network activity and gene expression of ANPEP are both mildly correlated to risk in patients that do not bear high-risk features or t(11;14) ($p_{expression}<1.4 \times 10^{-2}$, $p_{activity}<1.7 \times 10^{-2}$). ANPEP network activity, but not gene expression, is also correlated to risk in t(14;16) patients ($p_{activity}<2.3 \times 10^{-3}$). The most noteworthy trend in ANPEP expression, however, is that it becomes more highly expressed at relapse ($p<7 \times 10^{-3}$), in accordance with the success of Melflufen in RRMM.[25]

Differential activity of drug targets at relapse

Both network activity and gene expression showed decreased activity of CRBN at relapse when pooling all baseline versus all first-relapse patients ($p_{expression}<1.5 \times 10^{-3}$, $p_{activity}<1.1 \times 10^{-2}$). However, only network activity showed a significant difference (p<0.05) in the pairwise comparison of CRBN in the 39 patients with matched data. The network activity of IKZF1 is also significantly higher at baseline than relapse ($p_{activity}<4.3 \times 10^{-4}$), but the expression of IKZF1 alone does not show significant difference ($p_{expression}>0.8$). Decreased CRBN activity at relapse suggests a possible mechanism of escape from IMiD therapy by limiting the substrate for IKZF1 degradation, whereas decreased IKZF1 activity at relapse suggests a possible mechanism of escape wherein the MM cells are no longer dependent upon the activity of IKZF1. The expression of CRBN and IKZF1 are well correlated at relapse (spearman R=0.40, $p<2.3 \times 10^{-3}$), suggesting that the two possible mechanisms are not occurring in distinct subsets of relapse patients (*i.e.*, IKZF1 is not lowly expressed when CRBN is highly expressed).

Supplementary References

1. Hofree M, Shen JP, Carter H, Gross A, Ideker T. Network-based stratification of tumor mutations. *Nature Methods*. 2013;10(11):1108–1115.
2. Szklarczyk D, Gable AL, Lyon D, et al. STRING v11: protein–protein association networks with increased coverage, supporting functional discovery in genome-wide experimental datasets. *Nucleic Acids Research*. 2019;47(D1):D607–D613.
3. Liu Z, Huang J, Zhong Q, et al. Network-based analysis of the molecular mechanisms of multiple myeloma and monoclonal gammopathy of undetermined significance. *Oncol Lett*. 2017;14(4):4167–4175.
4. Robinson MD, Oshlack A. A scaling normalization method for differential expression analysis of RNA-seq data. *Genome Biol*. 2010;11(3):R25.
5. Huynh-Thu VA, Irrthum A, Wehenkel L, Geurts P. Inferring Regulatory Networks from Expression Data Using Tree-Based Methods. *PLOS ONE*. 2010;5(9):e12776.
6. Aibar S, González-Blas CB, Moerman T, et al. SCENIC: single-cell regulatory network inference and clustering. *Nature Methods*. 2017;14(11):1083–1086.
7. Margolin AA, Nemenman I, Basso K, et al. ARACNE: An Algorithm for the Reconstruction of Gene Regulatory Networks in a Mammalian Cellular Context. *BMC Bioinformatics*. 2006;7(S1):S7.
8. Faith JJ, Hayete B, Thaden JT, et al. Large-Scale Mapping and Validation of Escherichia coli Transcriptional Regulation from a Compendium of Expression Profiles. *PLOS Biology*. 2007;5(1):e8.
9. Reiss DJ, Baliga NS, Bonneau R. Integrated biclustering of heterogeneous genome-wide datasets for the inference of global regulatory networks. *BMC Bioinformatics*. 2006;7(1):280.
10. Brooks AN, Reiss DJ, Allard A, et al. A system-level model for the microbial regulatory genome. *Mol Syst Biol*. 2014;10(7):740.
11. Plaisier CL, O'Brien S, Bernard B, et al. Causal Mechanistic Regulatory Network for Glioblastoma Deciphered Using Systems Genetics Network Analysis. *Cell Systems*. 2016;3(2):172–186.
12. Neph S, Vierstra J, Stergachis AB, et al. An expansive human regulatory lexicon encoded in transcription factor footprints. *Nature*. 2012;489(7414):83–90.
13. Aten JE, Fuller TF, Lusis AJ, Horvath S. Using genetic markers to orient the edges in quantitative trait networks: The NEO software. *BMC Systems Biology*. 2008;2(1):34.
14. Wang L, Michoel T. Efficient and accurate causal inference with hidden confounders from genome-transcriptome variation data. *PLOS Computational Biology*. 2017;13(8):e1005703.
15. Mason MJ, Schinke C, Eng CLP, et al. Multiple Myeloma DREAM Challenge Reveals Epigenetic Regulator *PHF19* As Marker of Aggressive Disease. Cancer Biology; 2019.
16. Kronke J, Udeshi ND, Narla A, et al. Lenalidomide Causes Selective Degradation of IKZF1 and IKZF3 in Multiple Myeloma Cells. *Science*. 2014;343(6168):301–305.
17. Dong Y, Poellinger L, Gustafsson J-Å, Okret S. Regulation of Glucocorticoid Receptor Expression: Evidence for Transcriptional and Posttranslational Mechanisms. *Molecular Endocrinology*. 1988;2(12):1256–1264.
18. Oerlemans R, Franke NE, Assaraf YG, et al. Molecular basis of bortezomib resistance: proteasome subunit β5 (PSMB5) gene mutation and overexpression of PSMB5 protein. *Blood*. 2008;112(6):2489–2499.
19. Neri P, Ren L, Gratton K, et al. Bortezomib-induced "BRCAness" sensitizes multiple myeloma cells to PARP inhibitors. *Blood*. 2011;118(24):6368–6379.
20. Reale A, Khong T, Mithraprabhu S, et al. TOP2A knockdown resensitizes carfilzomib-resistant HMCLs to carfilzomib. *Clinical Lymphoma, Myeloma and Leukemia*. 2015;15:e68.

21. Franqui-Machin R, Hao M, Bai H, et al. Destabilizing NEK2 overcomes resistance to proteasome inhibition in multiple myeloma. *Journal of Clinical Investigation*. 2018;128(7):2877–2893.
22. Turner JG, Dawson JL, Grant S, et al. Treatment of acquired drug resistance in multiple myeloma by combination therapy with XPO1 and topoisomerase II inhibitors. *J Hematol Oncol*. 2016;9(1):73.
23. Punnoose EA, Leverson JD, Peale F, et al. Expression Profile of BCL-2, BCL-XL, and MCL-1 Predicts Pharmacological Response to the BCL-2 Selective Antagonist Venetoclax in Multiple Myeloma Models. *Molecular Cancer Therapeutics*. 2016;15(5):1132–1144.
24. Kumar S, Kaufman JL, Gasparetto C, et al. Efficacy of venetoclax as targeted therapy for relapsed/refractory t(11;14) multiple myeloma. *Blood*. 2017;130(22):2401–2409.
25. Richardson PG, Bringhen S, Voorhees P, et al. Melflufen plus dexamethasone in relapsed and refractory multiple myeloma (O-12-M1): a multicentre, international, open-label, phase 1–2 study. *The Lancet Haematology*. 2020;
